# Rhodapentalenes: Pincer Complexes with Internal Aromaticity

**DOI:** 10.1016/j.isci.2019.08.027

**Published:** 2019-08-22

**Authors:** Qingde Zhuo, Hong Zhang, Linting Ding, Jianfeng Lin, Xiaoxi Zhou, Yuhui Hua, Jun Zhu, Haiping Xia

**Affiliations:** 1 State Key Laboratory of Physical Chemistry of Solid Surfaces and Collaborative Innovation Center of Chemistry for Energy Materials (iChEM), College of Chemistry and Chemical Engineering, Xiamen University, Xiamen 361005, China; 2 Department of Chemistry, Shenzhen Grubbs Institute, Southern University of Science and Technology, Shenzhen 518055, China

**Keywords:** Chemistry, Inorganic Chemistry, Organometallic Chemistry

## Abstract

Pincer complexes are a remarkably versatile family benefited from their stability, diversity, and tunability. Many of them contain aromatic organic rings at the periphery, and aromaticity plays an important role in their stability and properties, whereas their metallacyclic cores are not aromatic. Herein, we report rhodapentalenes, which can be viewed as pincer complexes in which the metallacyclic cores exhibit considerable aromatic character. Rhodapentalenes show good thermal stability, although the rhodium-carbon bonds in such compounds are fragile. Experimental and computational studies suggest that the stabilization of rigid CCC pincer architectures together with an intrinsic aromaticity is vital for these metallacyclic rhodium species. Dearomatization-aromatization reactions, corresponding to metal-ligand cooperation of classical aromatic pincer complexes, were observed in this system. These findings suggest a new concept for pincer chemistry, the internal aromaticity involving metal *d*-orbitals, which would be useful for exploiting the nature of construction motif and inspire further applications.

## Highlights

• A concept for pincer chemistry: internal aromaticity involving metal *d*-orbitals
• Stable cyclic rhodium complexes with obvious rhodium carbene character
• Dual stabilization of rigid CCC pincer architectures and intrinsic aromaticity
• Rhodium-containing aromatics with a single metal entity

## Graphical Abstract

**Figure:**
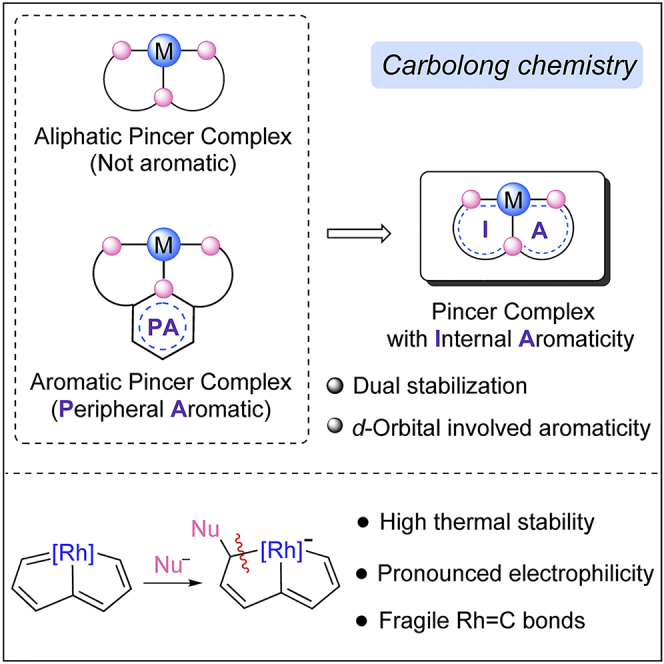

## Introduction

**Scheme 1:**
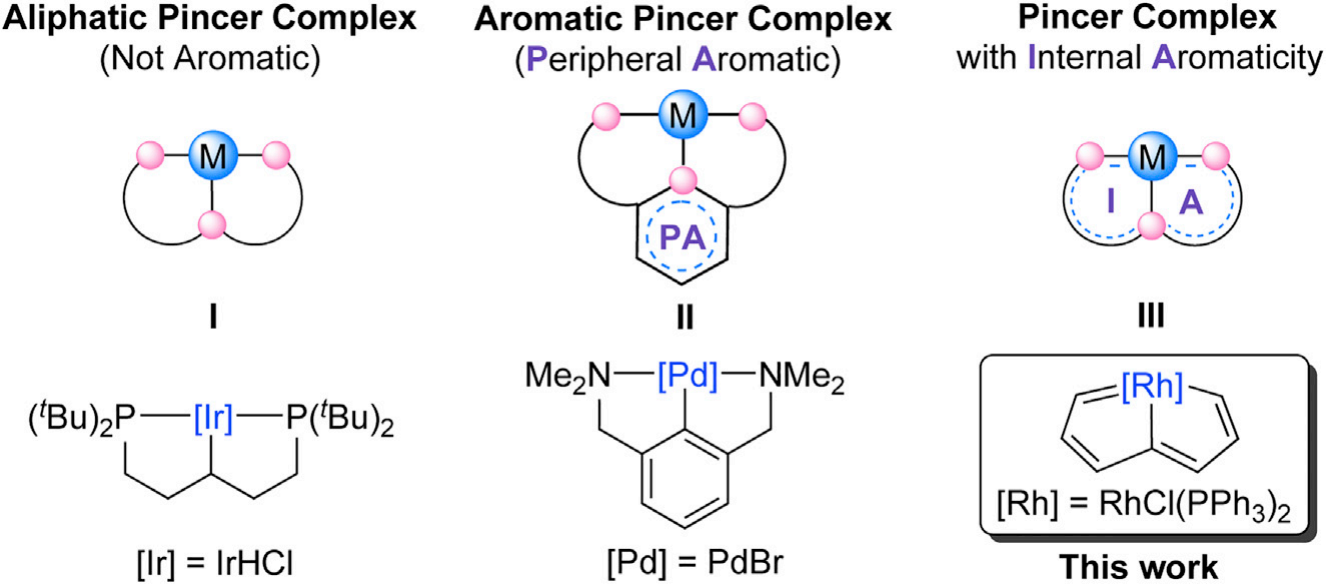
Primary Coordination Environment and New Concept of Pincer Complexes

Since the first reports in the late 1970s (Kelly et al., 1971, Moulton and Shaw, 1976, van Koten et al., 1978), pincer complexes have made great progresses and have been widely used in the fields of synthesis (Martinez et al., 2016, Pell et al., 2017, Rafiq et al., 2019), catalysis (Gao et al., 2018, Luque-Urrutia et al., 2019, Nielsen et al., 2013, Niu et al., 2019, Zhang et al., 2018), materials science (Albrecht et al., 2000, To et al., 2017, Zhang et al., 2017a), and biological systems (Desguin et al., 2015, Fellner et al., 2017). This has had an effect on the development of inorganic chemistry, materials chemistry, supramolecular chemistry, and bioorganometallics chemistry and on bond-making and bond-breaking processes (Albrecht and Lindner, 2011, Albrecht and van Koten, 2001, Gunanathan and Milstein, 2014, Kumar et al., 2017, Leis et al., 2008, Li et al., 2019, Morales-Morales, 2018, O’Reilly and Veige, 2014, Szabó and Wendt, 2014). Pincer complexes typically refer to tridentate chelates where the tridentate ligands bind to the metal centers in a meridional fashion (Albrecht and van Koten, 2001, Peris and Crabtree, 2018). The advantages of pincer complexes lie in their good thermal stability and tunable chemical properties. The electronic and steric properties of the metal centers can be easily modulated by introducing donor atoms (N, O, P, S, and C, etc.), substituents (alkyl or aryl groups), and frameworks (neutral, anionic, and cationic frameworks, etc.) and by the size of the metallic rings (five- or six-membered rings) of the pincer complexes. The thermal stability of pincer complexes can be enhanced by increasing the rigidity of pincer ligands, and a common way to achieve rigid pincer ligands is to incorporate the aromatic groups into the ligand backbones (Peris and Crabtree, 2018). Thus, although both aliphatic and aromatic backbones, represented by **I** and **II** in Scheme 1, were introduced in the early work of pincer complexes (Empsall et al., 1977, Kelly et al., 1971, Moulton and Shaw, 1976, van Koten et al., 1978), the latter, exemplified by the aromatic NCN palladium pincer complex (van Koten et al., 1978) shown in Scheme 1 have dominated the subsequent literature.

Aromaticity is one of the most fundamental concepts in chemistry, and has played an important role in the development of pincer chemistry. Aromatic pincer ligands provide a rigid coordination environment for the metal center, thus resulting in pincer complexes with high thermal stability (Albrecht and van Koten, 2001). New mode of metal-ligand cooperation was developed based on the dearomatization-aromatization of pincer ligands, which has led to unusual bond activation processes for many novel environmentally benign catalysis (Gunanathan and Milstein, 2011, Gunanathan and Milstein, 2013, Gunanathan and Milstein, 2014, Khusnutdinova and Milstein, 2015, Li et al., 2019, Zell and Milstein, 2015). A general feature of these pincer complexes is the aromatic organic rings fused to their central metallacycles, whereas the metallacycles of these pincer complexes are not aromatic. Herein we refer to these complexes as “pincer complexes with peripheral aromaticity” (Scheme 1, middle).

In this article, we describe rhodapentalenes, a class of compounds that can be considered as pincer complexes, and present a concept of pincer complex with internal aromaticity (Scheme 1, right). Unlike traditional pincer complexes in which the metal centers are not involved in the aromatic rings, the metal centers of pincer complexes with internal aromaticity participate in the construction of the aromatic systems and the metallacyclic core is aromatic. The rhodium pincer complexes with internal aromaticity, rhodapentalenes, were synthesized by efficient one-pot reactions of triyne chains with commercially available RhCl(PPh_3_)_3_ and acid. Remarkably, although rhodapentalenes display an evident rhodium carbene character and readily undergo ring-opening and ring-expansion reactions with nucleophiles and oxidants, respectively, they exhibit good thermal stability. Experimental and theoretical studies show that the intrinsic aromaticity together with the rigid pincer frameworks stabilize the cyclic rhodium species. Notably, dearomatization-aromatization processes related to classical aromatic pincer complexes were also developed in the rhodapentalene system.

## Results

### Synthesis and Characterization of CCC Rhodium Pincer Complexes: Rhodapentalenes

**Scheme 2:**
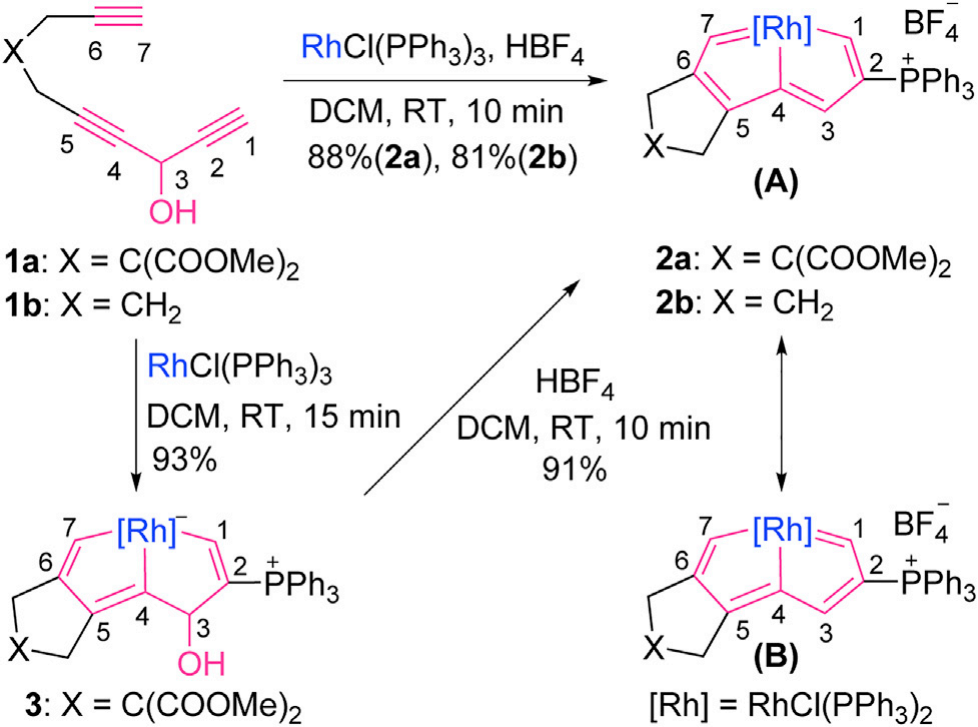
Synthesis of Rhodapentalenes

As shown in Scheme 2, treatment of the triyne chain **1a** or **1b** (Zhuo et al., 2017) with RhCl(PPh_3_)_3_ and HBF_4_ at room temperature (rt) led to the rhodapentalenes **2a** or **2b**, CCC rhodium pincer complexes, in isolated yields of 88% and 81%, respectively. The structures of **2a** and **2b** were confirmed by NMR spectroscopy (please see Figures S18–S37 for the NMR spectra of the reported compounds in this work). Their NMR spectra are similar; in the ^1^H NMR spectrum of **2a**, C^1^*H*, C^3^*H,* and C^7^*H* were observed at 11.76, 7.91, and 12.83 ppm and in the spectrum of **2b,** at 11.50, 8.07, and 13.08 ppm, respectively. These proton chemical shifts are consistent with those of the reported osmapentalenes (Zhu et al., 2014) and osmapentalynes (Zhu et al., 2013, Zhu and Xia, 2018, Zhuo et al., 2017) and are located in the metalla-aromatic region (Bleeke, 2001, Cao et al., 2014, Chen and Jia, 2013; Fernández et al., 2015, Frogley and Wright, 2018, Landorf and Haley, 2006, Saito, 2012, Wei et al., 2018, Wright, 2017). In the ^13^C NMR spectra, the resonances of the metal-bound carbon atoms appeared at 239.66 (C1), 188.66 (C4), and 265.29 ppm (C7) for **2a** and at 239.86 (C1), 187.62 (C4), and 261.20 ppm (C7) for **2b**. The chemical shifts of C4 are slightly down-field from those of Rh–C_vinyl_ carbons and fall within the range of Rh–C_aryl_ carbons (Kubo et al., 2005), whereas the chemical shifts of C1 and C7 are consistent with those reported for rhodium carbenes (Kornecki et al., 2013, Werlé et al., 2016).

**Figure 1:**
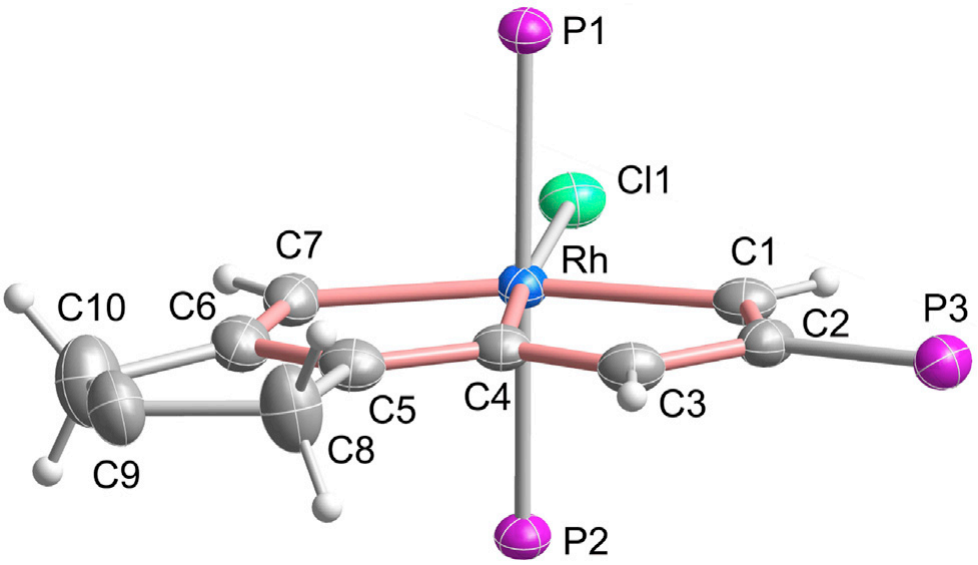
X-Ray Molecular Structure of the Cation of Rhodapentalene **2a** Ellipsoids are displayed at the 50% probability. The phenyl groups of PPh_3_ and the ester groups on C9 are omitted for clarity. Selected bond distances (Å) and bond angles (deg): Rh–C1 2.048(3), Rh–C4 1.987(3), Rh–C7 2.056(3), C1–C2 1.372(5), C2–C3 1.425(5), C3–C4 1.362(5), C4–C5 1.405(5), C5–C6 1.387(5), C6–C7 1.399(5); Rh–C1–C2 113.9(3), C1–C2–C3 115.3(3), C2–C3–C4 114.5(3), C3–C4–Rh 116.8(2), C1–Rh–C4 79.39(13), Rh–C4–C5 114.9(2), C4–C5–C6 116.2(3), C5–C6–C7 115.1(3), C6–C7–Rh 113.5(2), C7–Rh–C4 80.14(13).

X-ray crystallographic analysis confirmed the pincer structure of **2a**. As shown in Figure 1 (see Figure S1 for details), the tridentate CCC pincer ligand is attached to the rhodium center. The core metallabicycle is coplanar, as indicated by the small mean deviation (0.018 Å) from the least-squares plane through Rh and C1 to C7. The carbon-carbon bond distances (1.362(5)–1.425(5) Å) in the metallabicycle core show somewhat alternation, but are intermediate between typical single and double bonds, which is comparable with those of typical metalla-aromatics, such as the first metallabenzene (1.36(2)–1.42(2) Å) (Elliott et al., 1982), metallabenzyne (1.376(5)–1.420(5) Å) (Wen et al., 2001), and osmapentalene (1.365(5)–1.414(9) Å) (Zhu et al., 2014). The bond lengths of Rh–C1 (2.048(3) Å) and Rh–C7 (2.056(3) Å) are identical and at the high end of the range for Rh=C double bonds (1.743–2.059 Å, ranges based on data from the Cambridge Structural Database, CSD version 5.40, in November 2018) (Werlé et al., 2016). The Rh–C4 bond length is 1.987(3) Å, which is slightly shorter than those of Rh–C1 and Rh–C7. This is possibly a combined result of the weak *trans* influence of the Cl ligand (Housecroft and Sharpe, 2012) and the delocalized structure of **2a** around the peripheral skeleton. The structural features of **2a** are similar to those of osmapentalenes (Zhu et al., 2014), suggesting that it can be represented by the resonance structures of rhodapentalene **A** and rhodapentalene **B** (Scheme 2). Note that although carbon is ubiquitous in coordination chemistry, carbon as binding atom is relatively rare and CCC rhodium pincer complexes are uncommon (Kubo et al., 2005). To our knowledge, rhodapentalenes **2** represent the first examples of all-carbon-ligated rhodium pincer complexes, in which the pincer skeletons are composed entirely of carbon atoms. Remarkably, rhodapentalenes **2** exhibit high thermal stability. For example, **2a** can survive in air at 120°C for at least 3 h in the solid state, and subsequent thermal gravimetric analysis shows that weight changes occur only over 150°C (see Figures S7 and S8).

**Figure 2:**
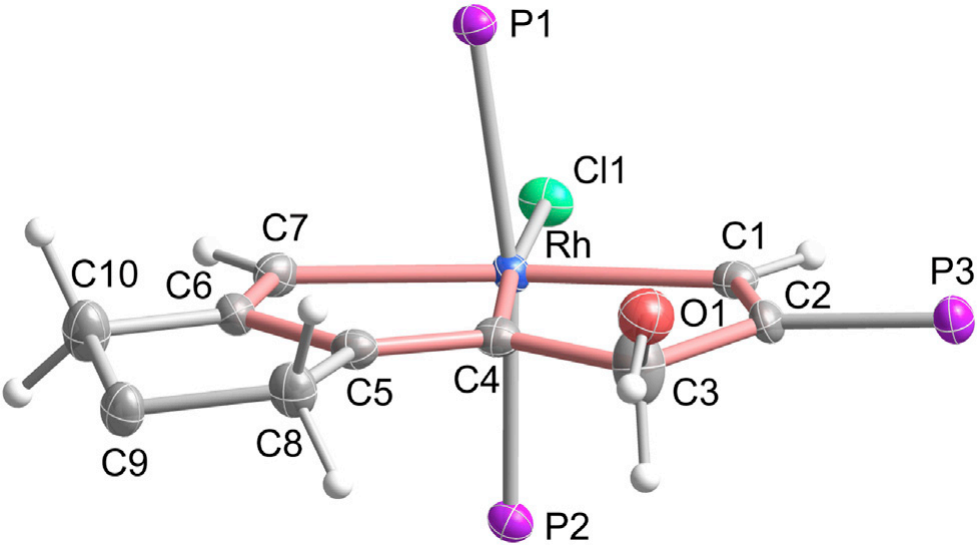
X-Ray Molecular Structure of Compound **3** Ellipsoids are displayed at the 50% probability. The phenyl groups of PPh_3_ and the ester groups on C9 are omitted for clarity. Selected bond distances (Å) and bond angles (deg): Rh–C1 2.065(4), Rh–C4 1.999(4), Rh–C7 2.118(4), C1–C2 1.358(6), C2–C3 1.503(6), C3–C4 1.479(6), C3–O1 1.395(6), C4–C5 1.375(6), C5–C6 1.461(6), C6–C7 1.337(6); Rh–C1–C2 115.4(3), C1–C2–C3 117.4(4), C2–C3–C4 108.9(4), C2–C3–O1 115.6(4), C3–C4–Rh 117.5(3), C1–Rh–C4 80.29(17), Rh–C4–C5 114.7(3), C4–C5–C6 115.8(4), C5–C6–C7 117.1(4), C6–C7–Rh 111.6(3), C7–Rh–C4 80.76(17).

To understand the mechanism for the formation of rhodapentalenes **2**, we studied the reaction of **1a** with RhCl(PPh_3_)_3_ in the absence of HBF_4_, which led to a new CCC pincer complex (**3**), isolated in 93% yield (Scheme 2). The structure of **3** was determined by X-ray crystallographic analysis (Figures 2 and S2). The crystal structure of **3** shows that C3 is *sp*^3^-hybridized and is attached to a hydroxyl group. Distinct from the delocalized structure of **2a**, the core structure in **3** is localized within the metallabicycle, as reflected by the bond lengths in the structure. The C1–C2 (1.358(6) Å), C4–C5 (1.375(6) Å), and C6–C7 (1.337(6) Å) bond lengths are consistent with those of double bonds, whereas the C2–C3 (1.503(6) Å), C3–C4 (1.479(6) Å), and C5–C6 (1.461(6) Å) bond lengths are appropriate for single bonds. The bond lengths of the three Rh–C bonds are 2.065(4) Å (Rh–C1), 1.999(4) Å (Rh–C4), and 2.118(4) Å (Rh–C7), which are in accordance with the bond lengths of Rh–C_vinyl_ bonds (Wu et al., 2002). The Rh–C1 and Rh–C7 bond lengths are obviously longer than the corresponding Rh–C bonds lengths in **2a**. In addition, **3** is less thermally stable than **2a**. A solid sample of **3** begins to convert into unidentified material at 60°C in air.

In view of the fact that the hydroxyl group is labile in the presence of acids, we conjectured that the rhodabicycle (**3**) may be a key intermediate in the formation of rhodapentalene (**2a**). As expected, when HBF_4_ was added to a green solution of **3**, the reaction mixture immediately turned red and **2a** was isolated in 91% yield after workup (Scheme 2). Based on the experimental observations, a plausible mechanism was postulated for the formation of **2** in Scheme S1.

### DFT Computations on the Internal Aromaticity of Pincer Complexes **2**

**Scheme 3:**
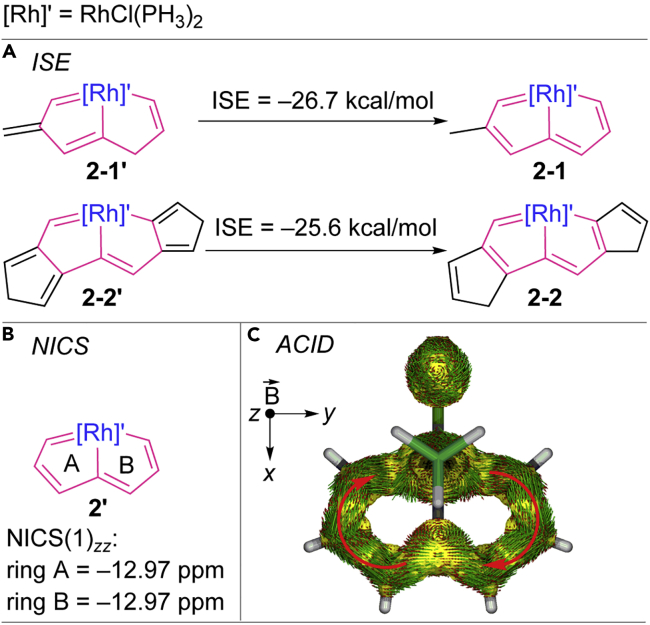
Evaluation of the Internal Aromaticity of Model Pincer Complex **2′** (A) ISE (isomerization stabilization energy) evaluations of the aromaticity of **2′**. (B) NICS(1)_*zz*_ (nucleus-independent chemical shift along the z axis at 1 Å above the ring critical point) evaluations of the aromaticity of **2′**. (C) ACID (anisotropy of the current-induced density) plot of **2′** from π contribution with an isosurface value of 0.025. The magnetic field vector is orthogonal to the ring plane and points upward, i.e., outward direction of the z axis (aromatic species exhibit clockwise diatropic circulations).

The good thermal stability, ring planarity, and low-field proton chemical shifts indicate that rhodapentalenes **2** are aromatic. To gain more insight into the aromaticity and electronic structure of the rhodapentalenes **2**, we performed density functional theory ([DFT] B3LYP/6-311++G(d,p)) calculations based on a simplified unsubstituted model complex **2′**, in which the PPh_3_ ligands were replaced by PH_3_ groups (see the cartesian coordinate in Data S2). We first investigated the aromatic stabilization energy of **2′** by the isomerization stabilization energy (ISE) method outlined by Schleyer and Pühlhofer (Schleyer and Pühlhofer, 2002, Wannere et al., 2003). As shown in Scheme 3A, the ISE values of **2′** (−26.7 and −25.6 kcal mol^−1^) are close to those of benzene (−33.2 and −29.0 kcal mol^−1^) (Schleyer and Pühlhofer, 2002, Wannere et al., 2003), indicating global aromaticity in **2**. The nucleus-independent chemical shift (NICS) is another computational method commonly exploited to diagnose aromaticity (Schleyer et al., 1996). The NICS(1)_*zz*_ value for each ring of **2′** was calculated to be −12.97 ppm (Scheme 3B, also see the values of the model complex **2-PH**_**3**_ with the charged phosphonium group in Figure S13), which is comparable with the value (−19.6 ppm) calculated by Mauksch and Tsogoeva (Mauksch and Tsogoeva, 2018), and indicates the aromaticity of **2**. The aromaticity of **2** is further supported by the anisotropy of the current-induced density (Herges and Geuenich, 2001), which can simulate the density and direction of the induced ring current in a molecular system under an external magnetic field. As shown in Scheme 3C and Figure S17, an obvious clockwise diatropic ring current can be observed in the π-system of **2′**, indicating π-aromaticity in the two fused five-membered rings. Therefore, experimental observations and the results of DFT calculations both demonstrate that rhodapentalenes (**2**) exhibit considerable aromatic character. The facile synthesis of **2** could be attributed to the internal aromaticity of **2** and could be considered to be an aromaticity-driven process. Notably, although rhodium has been demonstrated to form the metalla-aromatic model complexes in the pioneering theoretical work of metalla-aromatics by Hoffmann (Thorn and Hoffmann, 1979), and is among the metals that have been most investigated in the theoretical studies of metalla-aromatic chemistry (Iron et al., 2003, Islas et al., 2014, Fernández and Frenking, 2007, Mauksch and Tsogoeva, 2018), well-defined rhoda-aromatics remain scarce (Wei et al., 2015, Zhang et al., 2017b). All the known examples require extra stabilization by coordination to additional metal fragments (Wei et al., 2015, Zhang et al., 2017b). Rhodapentalenes **2** represent the first free rhoda-aromatics.

**Figure 3:**
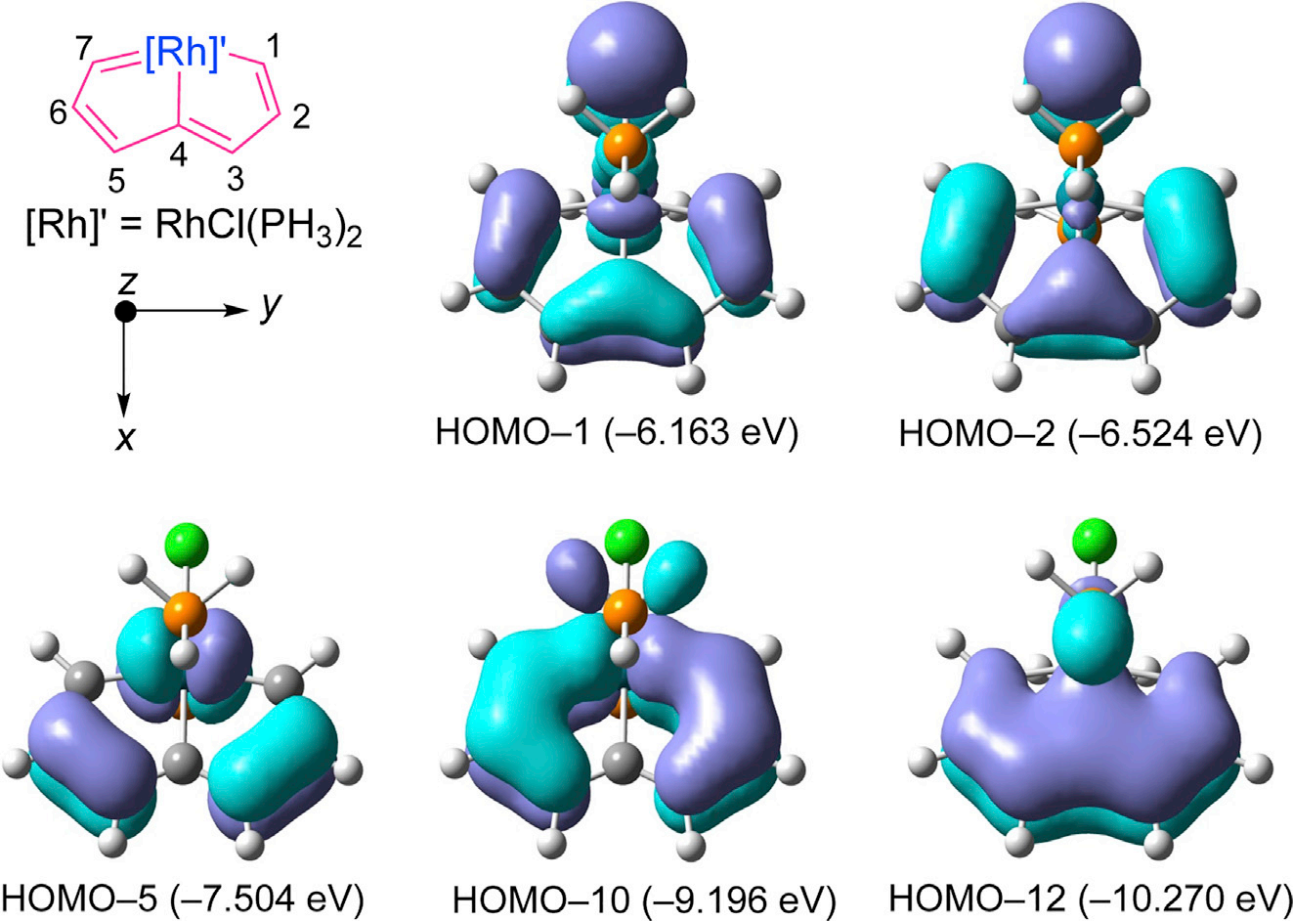
Selected Molecular Orbitals of **2′** The eigenvalues of the molecular orbitals are given in parentheses.

Selected molecular orbitals (MOs) for the model complex **2′** are shown in Figure 3. The five occupied π-MOs (highest occupied molecular orbital [HOMO]-1, HOMO-2, HOMO-5, HOMO-10, and HOMO-12) are derived mainly from the orbital interactions between the *d*-orbitals of the Rh atom (5*d*_*xz*_ and 5*d*_*yz*_) and the *p*_*zπ*_ orbitals of the C_7_H_5_ unit, indicating the involvement of the *d*-orbitals of the metal center in the π-delocalization along the perimeter of the pincer skeleton. These results are similar to those computed for the osmapentalenes (Zhu et al., 2014) and ruthenapentalenes (Zhuo et al., 2018). However, π-overlaps between the *d*-orbitals on metal centers and the *p*-orbital on the carbon atoms of rhodapentalene **2′** are less effective when compared with osmapentalenes (see Figures S9 and S10) and ruthenapentalenes (see Figures S11 and S12). To reveal the nature of the bonding in rhodapentalenes, natural bond orbital analysis was performed. The Wiberg bond indices ([WBIs] bond orders, which are a measure of bond strength) of C–C bonds for **2′** are between 1.33 and 1.56 and are in accordance with the unsaturated character of the rhodapentalene ring. Notably, the WBIs of Rh–C bonds (Rh–C1/C7: 0.76) are significantly smaller than those of Os–C (Os–C1/C7: 1.02) and Ru–C (Ru–C1/C7: 0.91) bonds in the corresponding metallapentalenes (see Table S3), which also confirms the diminished orbital hybridization of the metal center and the carbon atoms in rhodapentalenes. The WBIs of Rh–C bonds in rhodapentalenes are in accordance with those of Rh–C bonds in reported polarized rhodium-carbene complexes, which were critical intermediates in rhodium-catalyzed reactions (Padwa et al., 2000, Sheehan et al., 1988). We expect that the relatively weaker rhodium-carbon bonds would result in rhodapentalenes having high reactivity.

### Reactivity of Pincer Complex **2a**

**Scheme 4:**
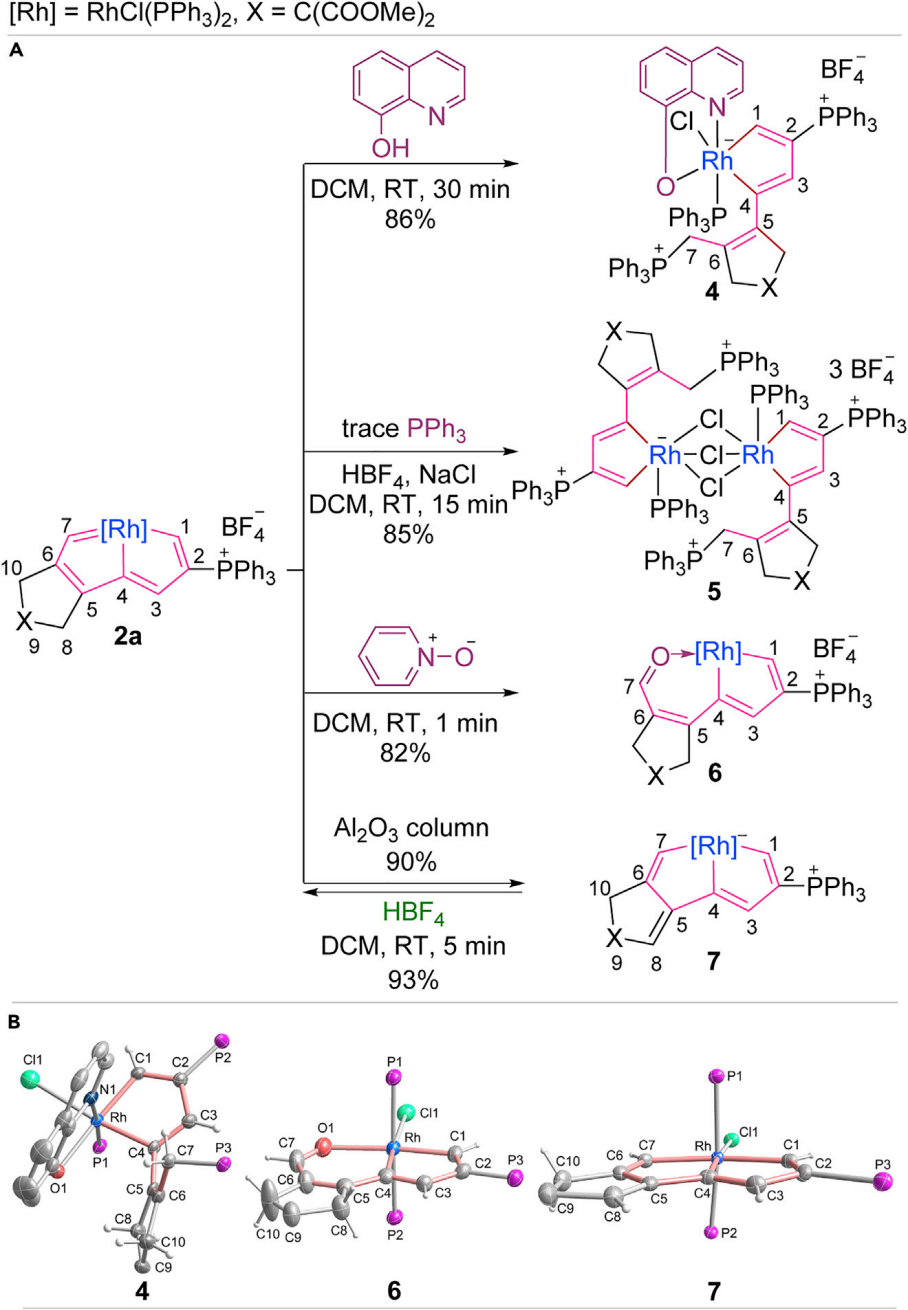
Reactions of Rhodapentalene **2a** (A) Ring-opening, ring-expansion, and dearomatization-aromatization reactions of rhodapentalene **2a** (B) X-ray molecular structure of complex **7** and the cations of complexes **4** and **6.** Ellipsoids are displayed at the 50% probability and the phenyl groups of PPh_3_, and the ester groups on C9 are omitted for clarity.

Encouraged by the computational results, we studied the reaction chemistry of rhodapentalenes. We chose 8-hydroxyquinoline to test the reactivity of rhodapentalene (**2a**) because of its versatile function as a bidentate ligand and a nucleophile. As shown in Scheme 4A, the reaction of **2a** with 8-hydroxyquinoline immediately produced a ring-opened product **4**, in which the Rh–C7 bond had been cleaved (see Scheme 4B and Figure S3 for its crystal structure). A possible mechanism is proposed in Scheme S2 for the formation of **4**.

Judging by the reaction of **2a** with 8-hydroxyquinoline, rhodapentalenes should be sensitive toward PPh_3_. Indeed, trace amounts of PPh_3_ can trigger the transformation of **2a** into a mixture, from which ring-opening product **5** can be isolated in low yield and whose X-ray crystal structure is shown in Figure S4. When the reaction was performed in the presence of HBF_4_ and NaCl, **5** can be obtained cleanly (Scheme 4A). We deduce that the formation of **5** (see Scheme S3) is also driven by a nucleophilic addition step, similar to that in the formation of **4**. It should be noted that nucleophilic addition-induced ring-opening reactions are rare in metalla-aromatic species (Zhang et al., 2010), and the reported examples usually require reaction temperatures above rt or a long reaction time (for example, more than 4 days) (Zhang et al., 2010). The mild reaction conditions in the case of **2a** could be attributed to the weakness of the rhodium-carbon bonds in rhodapentalenes, in agreement with the computational results.

We also probed the reactions of rhodapentalene **2a** with a number of oxidants, such as pyridine *N*-oxide, dimethyl sulfoxide, and 3-chloroperoxybenzoic acid, which all lead to the same product. As demonstrated by the reaction with pyridine *N*-oxide in Scheme 4A, **6** was isolated as a blue powder in 82% yield. The X-ray diffraction study revealed that **6** contains an oxygen-containing six-membered ring arising from the insertion of an oxygen atom into one of the five-membered metallacycles (see Scheme 4B and Figure S5). To our knowledge, there are no reports of oxygen-insertion reactions of metalla-aromatics. The observed ring expansion of rhodapentalene **2a** may be induced by the nucleophilic attack of pyridine *N*-oxide at the C7 site (see Scheme S4).

The reactions of **2a** with 8-hydroxyquinoline, PPh_3_, and pyridine *N*-oxide indicate the pronounced electrophilicity and lability of the rhodium-carbon bonds in rhodapentalenes and are consistent with the chemical behavior of rhodium carbene moieties (Berry, 2012, Tindall et al., 2018, Zhang et al., 2019). The steric crowding of the bulky phosphonium group adjacent to the C1-position may account for the C7-position being the sole active site in the reactions of **2a**.

Dearomatization-aromatization reactions, similar to those of classical pincer ligand systems (Gunanathan and Milstein, 2011, Gunanathan and Milstein, 2013, Gunanathan and Milstein, 2014, Khusnutdinova and Milstein, 2015, Li et al., 2019, Zell and Milstein, 2015), can also be achieved in rhodapentalenes. As shown in Scheme 4A, one of the protons attached to C8 in **2a** can be readily removed when operated in column chromatography with neutral alumina, leading to compound **7**. Both structural characterization data (Scheme 4B and Figure S6) and DFT calculations (Figures S14–S16) demonstrate the nonaromaticity of **7**. Interestingly, **7** could be re-aromatized upon treatment with HBF_4_, regenerating the original rhodapentalene **2a**. The interconversion between aromatic complex **2a** and nonaromatic complex **7** corresponds to the intriguing dearomatization-aromatization process of classical aromatic pincer complexes. Such dearomatization-aromatization reactions of pincer complexes based on pyridine and acridine have been regarded as promising activation mode via metal-ligand cooperation and led to extraordinary applications in various dehydrogenative/hydrogenative and bond activation reactions (Gunanathan and Milstein, 2011, Gunanathan and Milstein, 2013, Gunanathan and Milstein, 2014, Khusnutdinova and Milstein, 2015, Li et al., 2019, Zell and Milstein, 2015).

## Discussion

The discovery of rhodapentalenes (**2**) featuring both rhodium carbene and aromatic character leads to interesting questions regarding the enhancement of the stability of the compounds. The high thermal stability of rhodapentalenes **2** is somewhat surprising, as complexes with obvious rhodium carbene property are generally labile. Rhodium carbene complexes have often been proposed as key intermediates of numerous rhodium-catalyzed reactions but have rarely been isolated (Berry, 2012, Davies and Manning, 2008, DeAngelis et al., 2016). Besides, such cyclic rhodium carbene complexes have not been reported to date, although rhodium was predicted to form rhodabenzenes in a pioneering theoretical work of metalla-aromatic chemistry (Thorn and Hoffmann, 1979). Previous studies indicate that rhodabenzenes decompose readily through carbene migration reactions (Wu et al., 2002, Haley, 2017), and we reasoned that the exceptional thermal stability of rhodapentalenes (**2**) can be attributed to the considerable aromatic character and rigid chelating properties of CCC pincer frameworks.

Aromaticity has long been regarded as the property that can efficiently stabilize a wide range of reactive compounds. Besides that, the high thermal stabilities associated with pincer complexes have been ascribed to the chelating and multidentate nature of pincer ligands. The CCC-pincer ligand-based rhodapentalene (**2**) is significant in that it represents the first example of rhodium pincer complexes with aromatic metallacycles. The fact that the rhodium carbene structures formed through the chelation of aliphatic carbon chains with the metal center were very stable prompted us to re-examine the structure of pincer complexes. We envision that pincer complexes with aromatic metallacycle cores should be classified as pincer complexes with internal aromaticity (demonstration in Scheme 1). The significant difference between the pincer complexes with internal aromaticity (**III**) and common aromatic pincer complexes (**II**) is the participation in the aromaticity of the *d*-orbitals of the metal centers. We have recently reported metallapentalenes and metallapentalynes with group 8 metal centers. However, in these complexes the metal-carbon multiple bonds are rather robust (Zhu et al., 2013, Zhu and Xia, 2018, Zhuo et al., 2018). In the case of rhodapentalenes **2**, the metallapentalene chemistry has been extended for the first time from group 8 to group 9 elements. The phenomenon of both high reactivity and good thermal stability of rhodapentalenes **2** made us aware of the dual stabilization of pincer framework and aromaticity in this system for the first time. More interestingly, dearomatization/aromatization reactions can also be realized in rhodapentalene system. In this context, previous reported metallapentalynes (Zhu et al., 2013, Zhu and Xia, 2018, Zhuo et al., 2018), metallapentalenes (Zhu et al., 2014), and other bridge-headed fused metalla-aromatic compounds (Frogley et al., 2018, Frogley and Wright, 2014, Frogley and Wright, 2017, Wang et al., 2012, Wang et al., 2013) could also be regarded as pincer complexes with internal aromaticity. We believe that these conceptually new pincer complexes, which combine the structural features of both aromatic compounds and rigid pincer complexes would provide new opportunities for pincer chemistry.

In summary, we have discovered a new class of metalla-aromatic compounds, the rhodapentalenes, which can be viewed as CCC pincer complexes with internal aromaticity. Rhodapentalenes exhibit good thermal stability, although electronic structure analysis and reactivity studies reveal the high reactivity of the Rh–C(1) and Rh–C(7) bonds toward nucleophiles. These results demonstrate the promising stabilization of internal aromatic pincer frameworks, which should be attributed to dual stabilization deriving from both rigid polydentate chelation and aromatic stabilization energy. Given the above-mentioned findings the pincer complex platform with internal aromaticity can serve as a new candidate for the stabilization of vulnerable species.

### Limitations of the Study

The reactions of rhodapentalenes with unsaturated species such as alkenes and alkynes were also tested, but the anticipated carbene insertion products were not observed.

## Methods

All methods can be found in the accompanying Transparent Methods supplemental file.

## Data and Code Availability

Crystallographic data were deposited in the Cambridge Crystallographic Data Centre (CCDC) under accession numbers CCDC: 1506335 (**2a**), 1506333 (**3**), 1848362 (**4**), 1848363 (**5**), CCDC-1848364 (**6**), and CCDC-1848365 (**7**). These data can be obtained free of charge from the Cambridge Crystallographic Date Centre via www.ccdc.cam.ac.uk/data_request/cif. CIF files for **2a** and **3–7** are also provided in Data S1.

## Supplemental Information

Document S1. Transparent Methods, Figures S1–S37, Schemes S1–S4, and Tables S1–S3

Data S1. CIFs, Related to Figures 1, 2 and Scheme 4

Data S2. Cartesian Coordinate, Related to Figure 3 and Scheme 3
