# Supplementary material for: Rhodapentalenes: Pincer Complexes with Internal Aromaticity
Source: iScience. 2019 Aug 22;19:1214–24. doi: 10.1016/j.isci.2019.08.027 (PMC6831826; doi:10.1016/j.isci.2019.08.027)
Supplement: Document S1. Transparent Methods, Figures S1–S37, Schemes S1–S4, and Tables S1–S3 [file mmc1.pdf]

ISCI, Volume *19*

Supplemental Information

Rhodapentalenes: Pincer Complexes with Internal Aromaticity

Qingde Zhuo, Hong Zhang, Linting Ding, Jianfeng Lin, Xiaoxi Zhou, Yuhui Hua, Jun Zhu, and Haiping Xia

Proposed Mechanisms

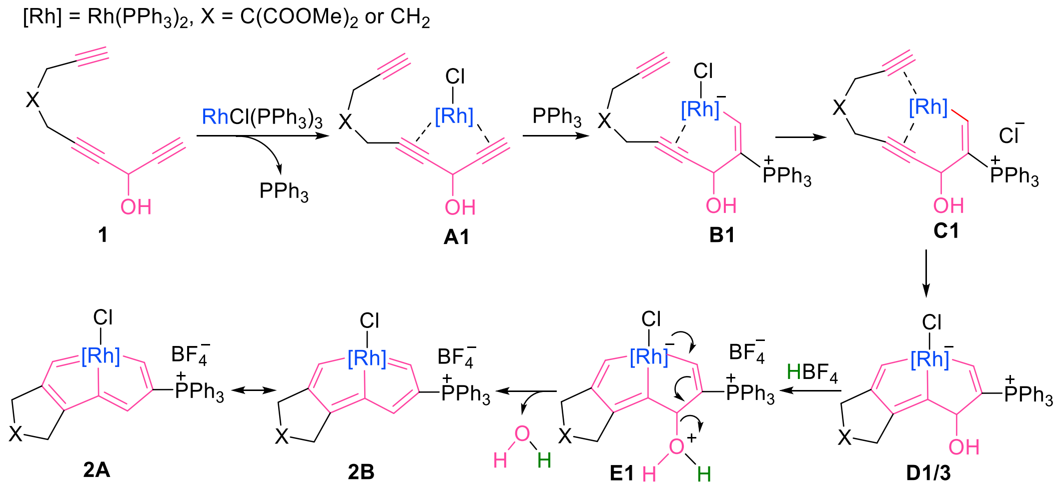

Scheme S1. Plausible mechanism for the formation of rhodapentalenes **2**, related to Scheme 2.

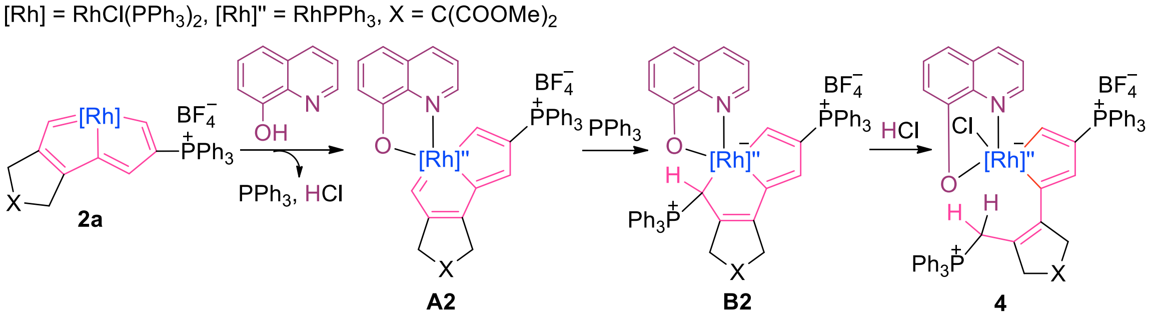

Scheme S2. Plausible mechanism for the formation of **4**, related to Scheme 4.

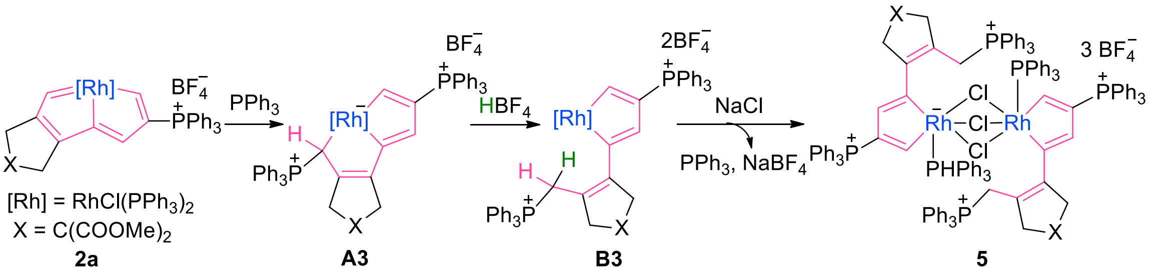

Scheme S3. Plausible mechanism for the formation of **5**, related to Scheme 4.

[Rh] = $RhCl(PPh_3)_2$, X = $C(COOMe)_2$

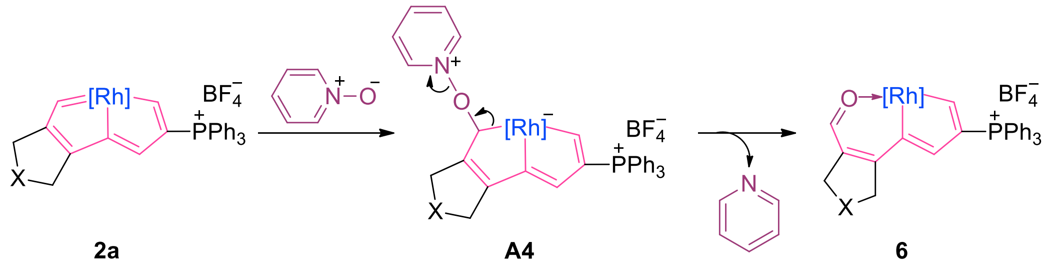

2a **A4** **6**

Scheme S4. Plausible mechanism for the formation of **6**, related to Scheme 4.

Crystal Structures

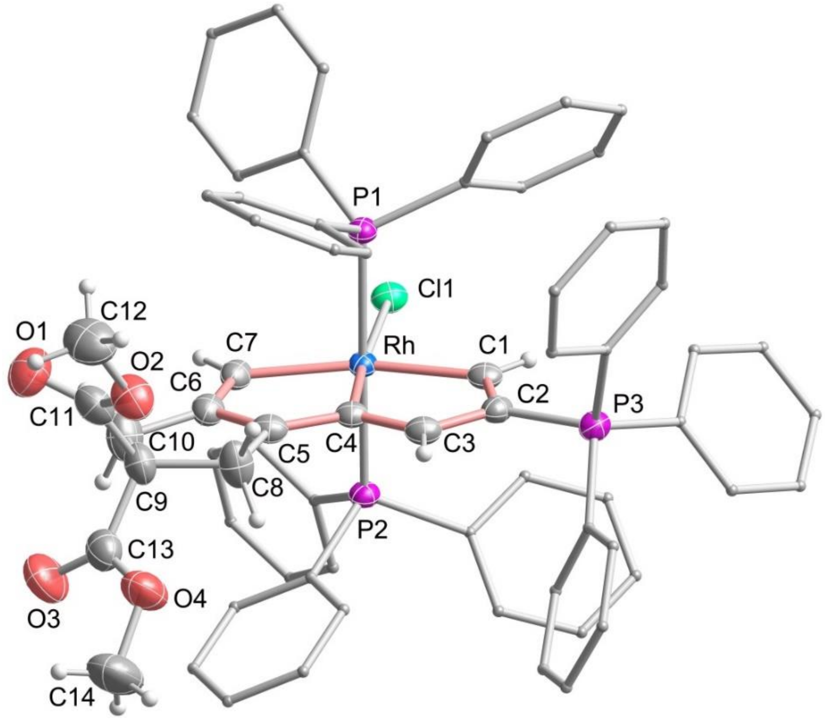

Figure S1. X-ray molecular structure of the cation of complex **2a** (ellipsoids are drawn at the 50% probability level). The hydrogen atoms of PPh_3 are omitted for clarity, related to Figure 1.

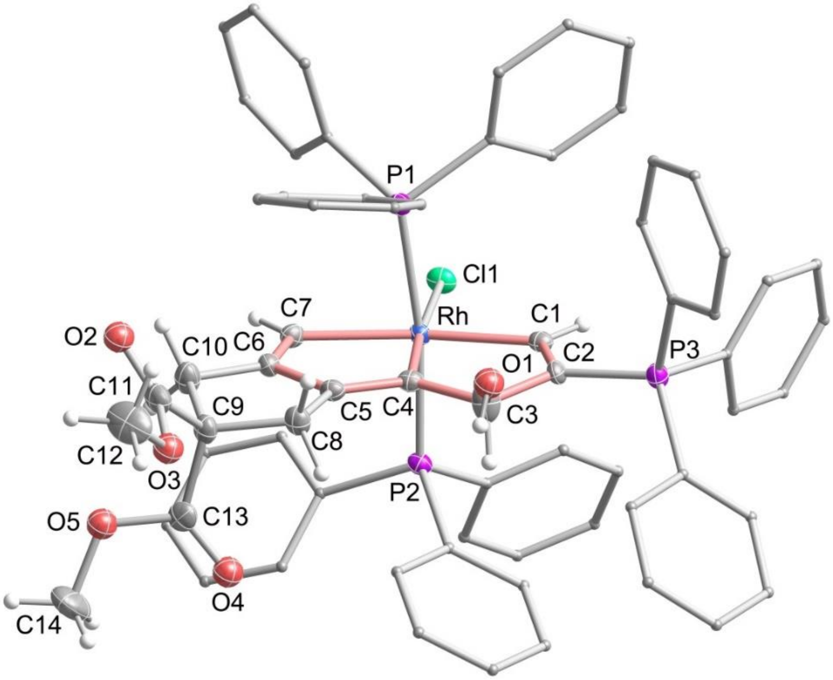

Figure S2. X-ray molecular structure of complex **3** (ellipsoids are drawn at the 50% probability level). The hydrogen atoms of PPh_3 are omitted for clarity, related to Figure 2.

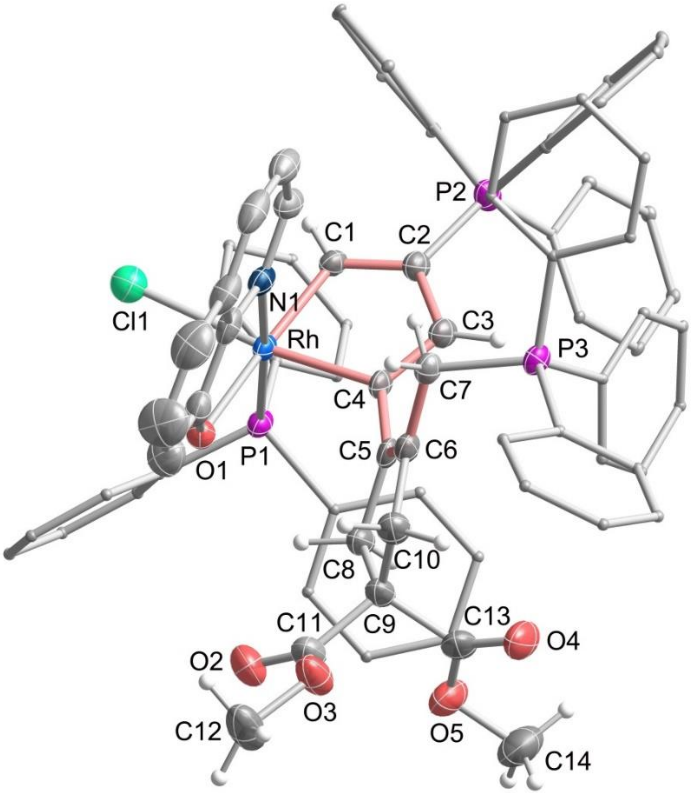

Figure S3. X-ray molecular structure of the cation of complex **4** (ellipsoids are drawn at the 50% probability level), related to Scheme 4B. The hydrogen atoms in the aromatic rings are omitted for clarity. Selected bond distances (Å) and angles (deg): Rh–C1 1.989(3), Rh–C4 2.038(3), C1–C2 1.358(4), C2–C3 1.465(4), C2–P2 1.781(3), C3–C4 1.346(5), C4–C5 1.470(4), C5–C6 1.347(5), C6–C7 1.499(5), C7–P3 1.826(3); Rh–C1–C2 115.0(2), C1–C2–C3 115.4(3), C2–C3–C4 114.2(3), C3–C4–Rh 114.4(2), C1–Rh–C4 80.45(12), Rh–C4–C5 118.5(2), C4–C5–C6 127.0(3), C5–C6–C7 125.2(3).

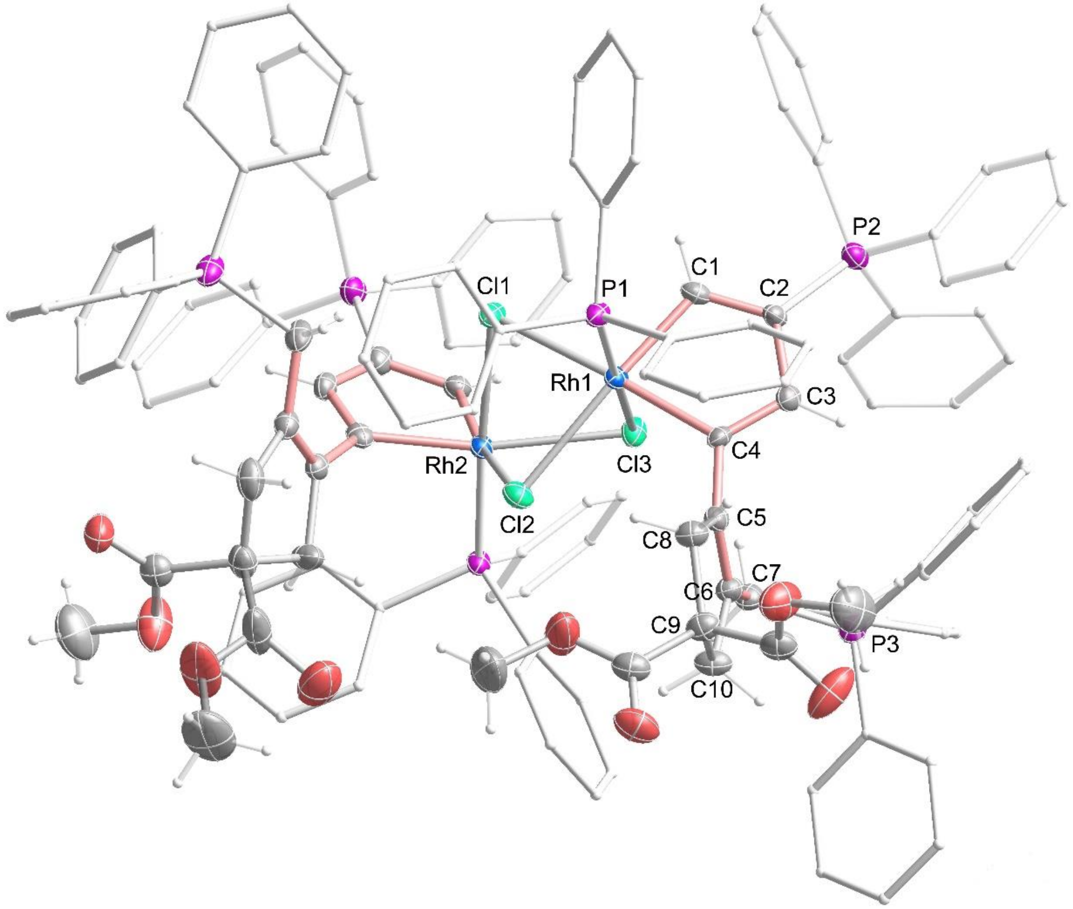

Figure S4. X-ray molecular structure of the cation of complex **5** (ellipsoids are drawn at the 50% probability level), related to Scheme 4. The hydrogen atoms of PPh_3 are omitted for clarity. Selected bond distances (Å) and angles (deg): Rh–C1 1.977(5), Rh–C4 2.022(5), C1–C2 1.351(7), C2–C3 1.469(7), C2–P2 1.776(5), C3–C4 1.348(7), C4–C5 1.454(7), C5–C6 1.348(7), C6–C7 1.492(7), C7–P3 1.822(5); Rh–C1–C2 115.4(4), C1–C2–C3 114.8(4), C2–C3–C4 114.2(4), C3–C4–Rh 114.2(4), C1–Rh–C4 80.7(2), Rh–C4–C5 121.0(3), C4–C5–C6 127.5(5), C5–C6–C7 125.3(5).

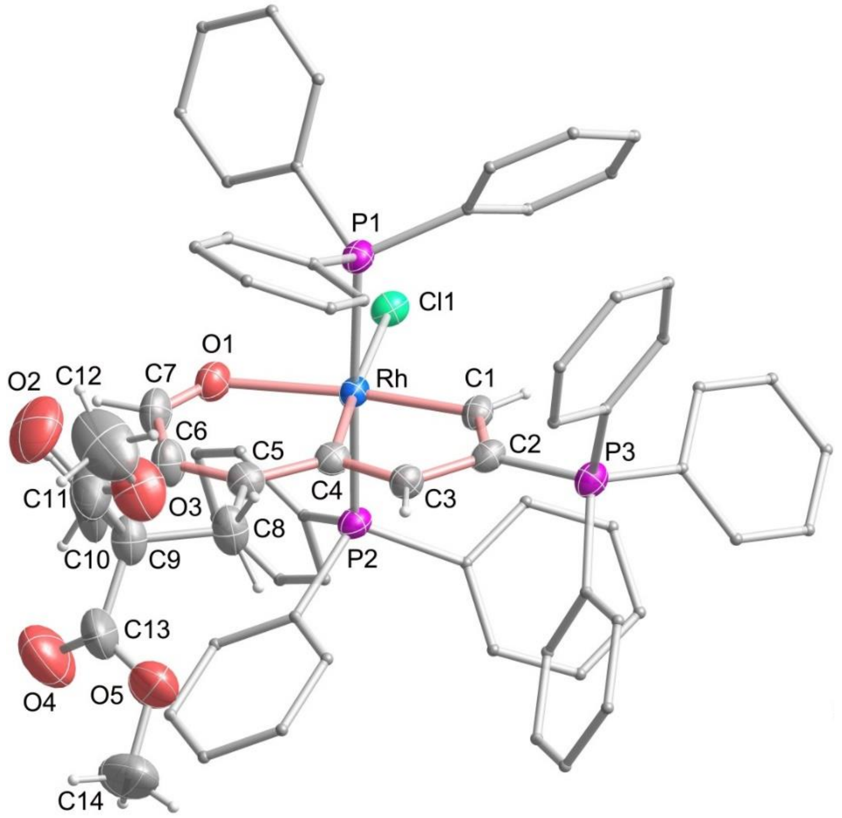

Figure S5. X-ray molecular structure of the cation of complex **6** (ellipsoids are drawn at the 50% probability level), related to Scheme 4B. The hydrogen atoms of PPh_3 are omitted for clarity. Selected bond distances (Å) and angles (deg): Rh–C1 1.982(4), Rh–C4 2.011(4), Rh–O1 2.182(3), C1–C2 1.350(6), C2–C3 1.449(6), C3–C4 1.350(6), C4–C5 1.437(6), C5–C6 1.352(6), C6–C7 1.437(7), C7–O1 1.225(5); Rh–C1–C2 115.7(3), C1–C2–C3 114.5(4), C2–C3–C4 114.9(4), C3–C4–Rh 114.4(3), C1–Rh–C4 80.41(16), Rh–C4–C5 122.8(3), C4–C5–C6 127.0(4), C5–C6–C7 127.3(4), C6–C7–O1 126.6(4), C7–O1–Rh1 125.1(3), O1–Rh–C4 90.96(14).

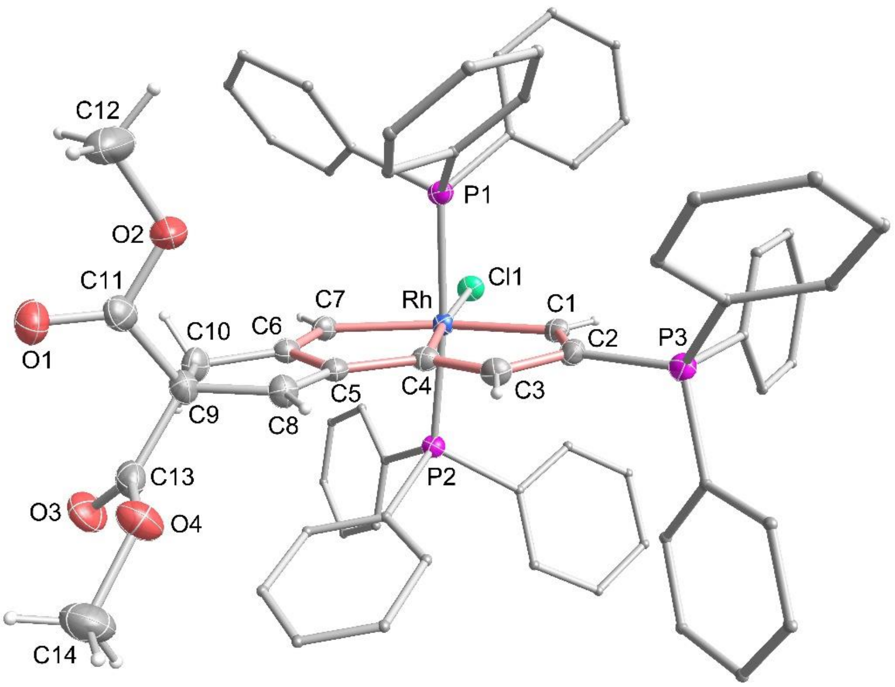

Figure S6. X-ray molecular structure of complex **7** (ellipsoids are drawn at the 50% probability level), related to Scheme 4B. The hydrogen atoms of PPh_3 are omitted for clarity. Selected bond distances (Å) and angles (deg): Rh–C1 2.056(3), Rh–C4 2.001(3), Rh–C7 2.114(3), C1–C2 1.365(5), C2–C3 1.459(5), C3–C4 1.338(5), C4–C5 1.472(5), C5–C6 1.451(5), C6–C7 1.342(5), C5–C8 1.346(5), C8–C9 1.521(5), C9–C10 1.573(5), C6–C10 1.516(5); Rh–C1–C2 112.9(2), C1–C2–C3 115.9(3), C2–C3–C4 114.5(3), C3–C4–Rh 116.6(2), C1–Rh–C4 80.10(13), Rh–C4–C5 113.3(2), C4–C5–C6 114.1(3), C5–C6–C7 118.5(3), C6–C7–Rh 112.3(2), C7–Rh–C4 81.83(13), C6–C5–C8 112.4(3), C5–C8–C9 110.9(3), C8–C9–C10 104.0(3), C9–C10–C6 104.5(3).

TGA Data for Complex 2a

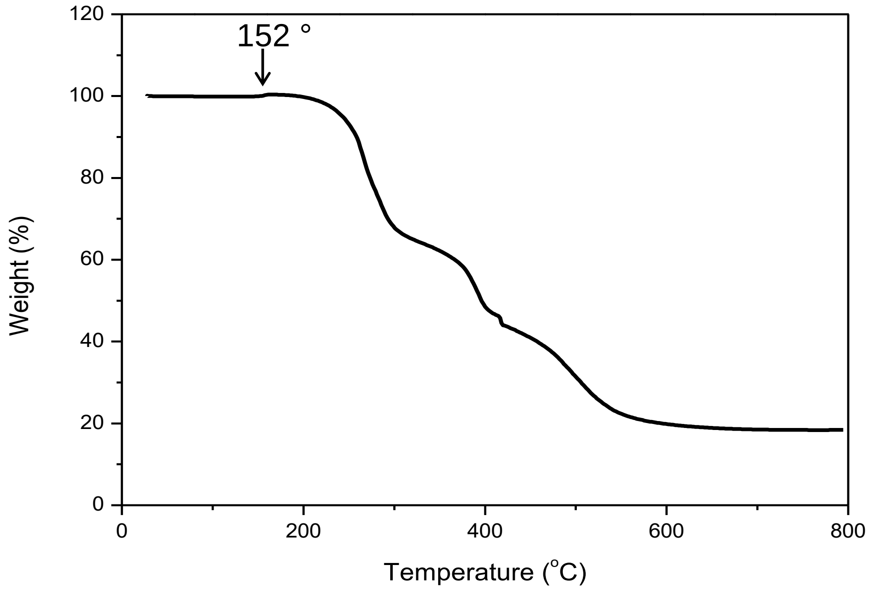

Figure S7. TGA data for complex **2a** at a constant heating rate of 10 °C min^{-1} under a flowing air atmosphere, related to Scheme 2.

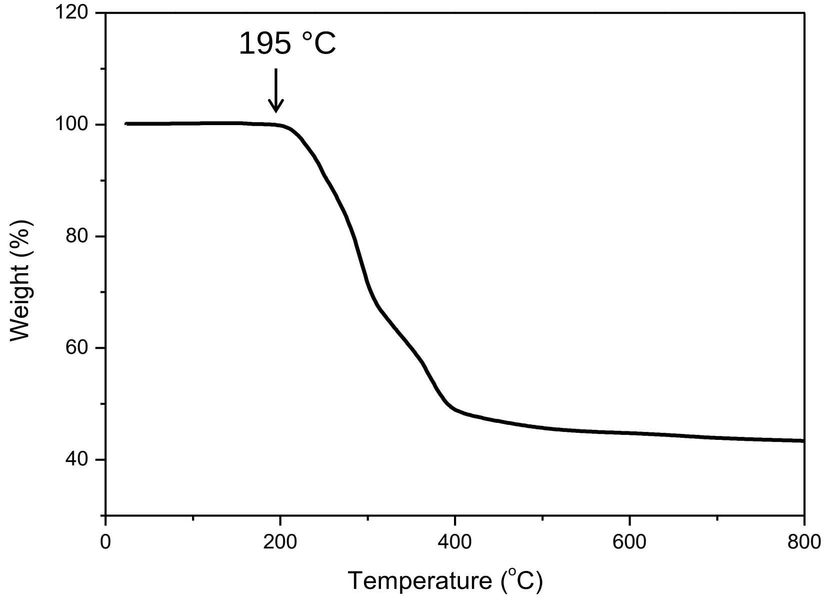

Figure S8. TGA data for complex **2a** at a constant heating rate of 10 °C min^{-1} under a flowing N_2 atmosphere, related to Scheme 2.

Selected Molecular Orbitals of Osmapentalenes and Ruthenapentalenes

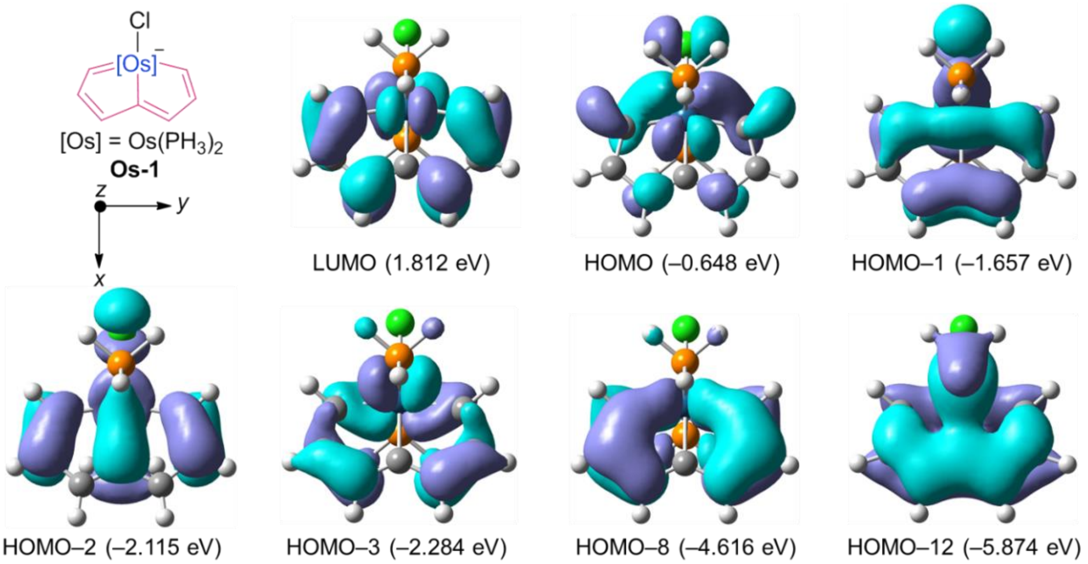

Figure S9. Selected frontier molecular orbitals of osmapentalene **Os-1** calculated at the B3LYP/[LanL2DZ for Os, Cl and P; 6-311++G(d,p) for C and H]. The eigenvalues of the molecular orbitals are given in parentheses, related to Figure 3.

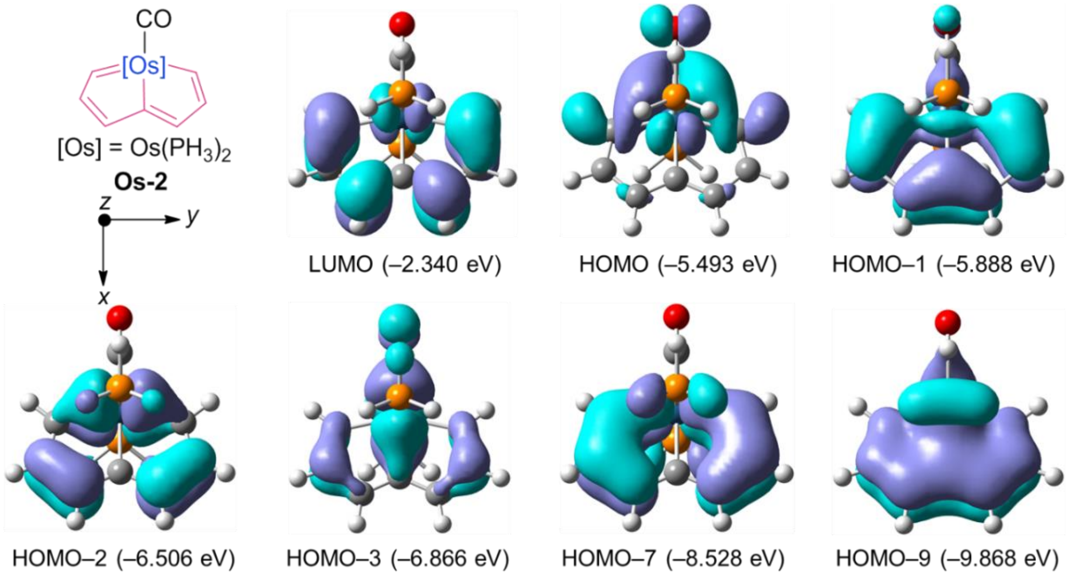

Figure S10. Selected frontier molecular orbitals of osmapentalene **Os-2** calculated at the B3LYP/[LanL2DZ for Os and P; 6-311++G(d,p) for O, C and H]. The eigenvalues of the molecular orbitals are given in parentheses, related to Figure 3.

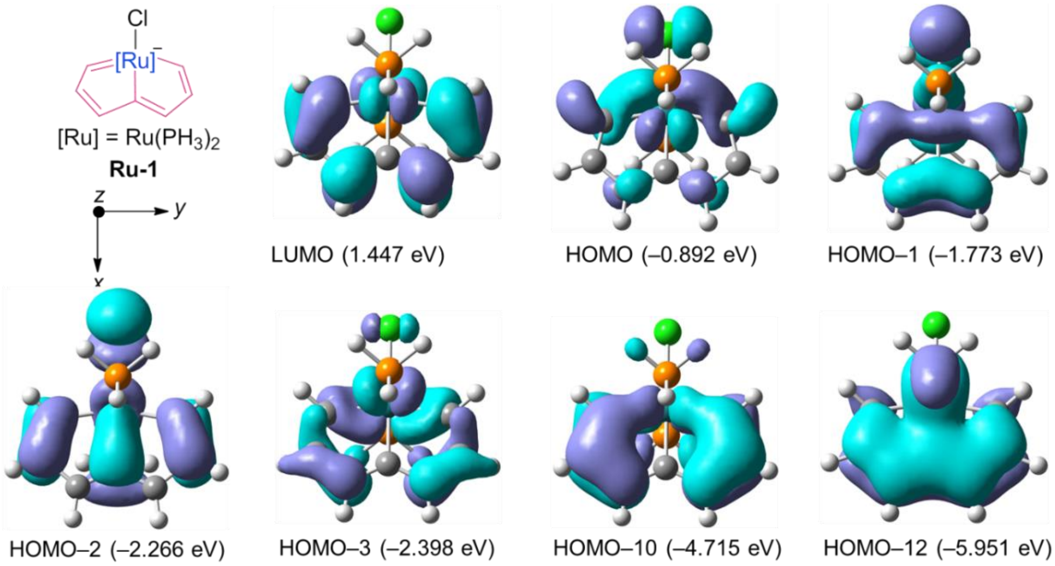

Figure S11. Selected frontier molecular orbitals of ruthenapentalene **Ru-1** calculated at the B3LYP/[LanL2DZ for Ru, Cl and P; 6-311++G(d,p) for C and H]. The eigenvalues of the molecular orbitals are given in parentheses, related to Figure 3.

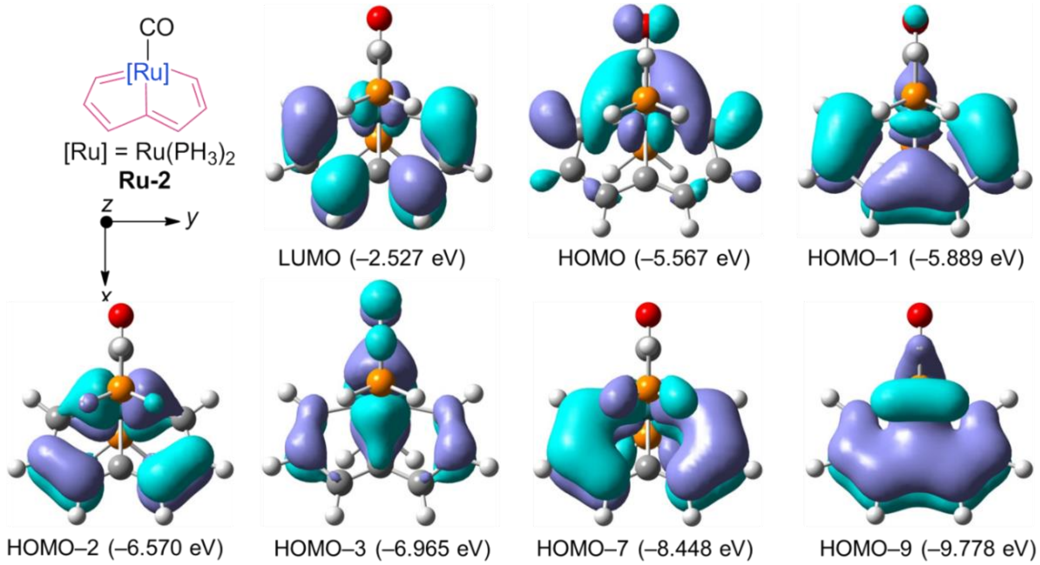

Figure S12. Selected frontier molecular orbitals of ruthenapentalene **Ru-2** calculated at the B3LYP/[LanL2DZ for Ru and P; 6-311++G(d,p) for O, C and H]. The eigenvalues of the molecular orbitals are given in parentheses, related to Figure 3.

Aromaticity Evaluations

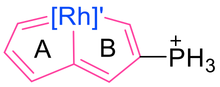

NICS(1)$_{zz}$:
ring A = –9.07 ppm
ring B = –11.63 ppm

[Rh]' = RhCl(PH$_3$)$_2$

2-PH$_3$

Figure S13. NICS evaluations of model complex **2-PH$_3$**, related to Scheme 3.

[Rh]' = RhCl(PH$_3$)$_2$

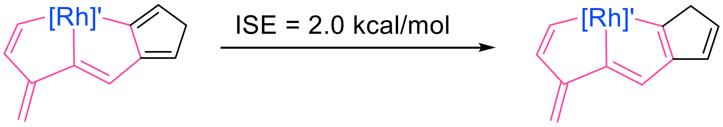

7-1' **7-1**

Figure S14. ISE evaluation of model complex **7-1'**, related to Scheme 4.

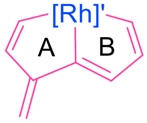

NICS(1)$_{zz}$:
ring A = –2.77 ppm
ring B= 2.05 ppm

[Rh]' = RhCl(PH$_3$)$_2$

7'

Figure S15. NICS evaluations of model complex **7'**, related to Scheme 4.

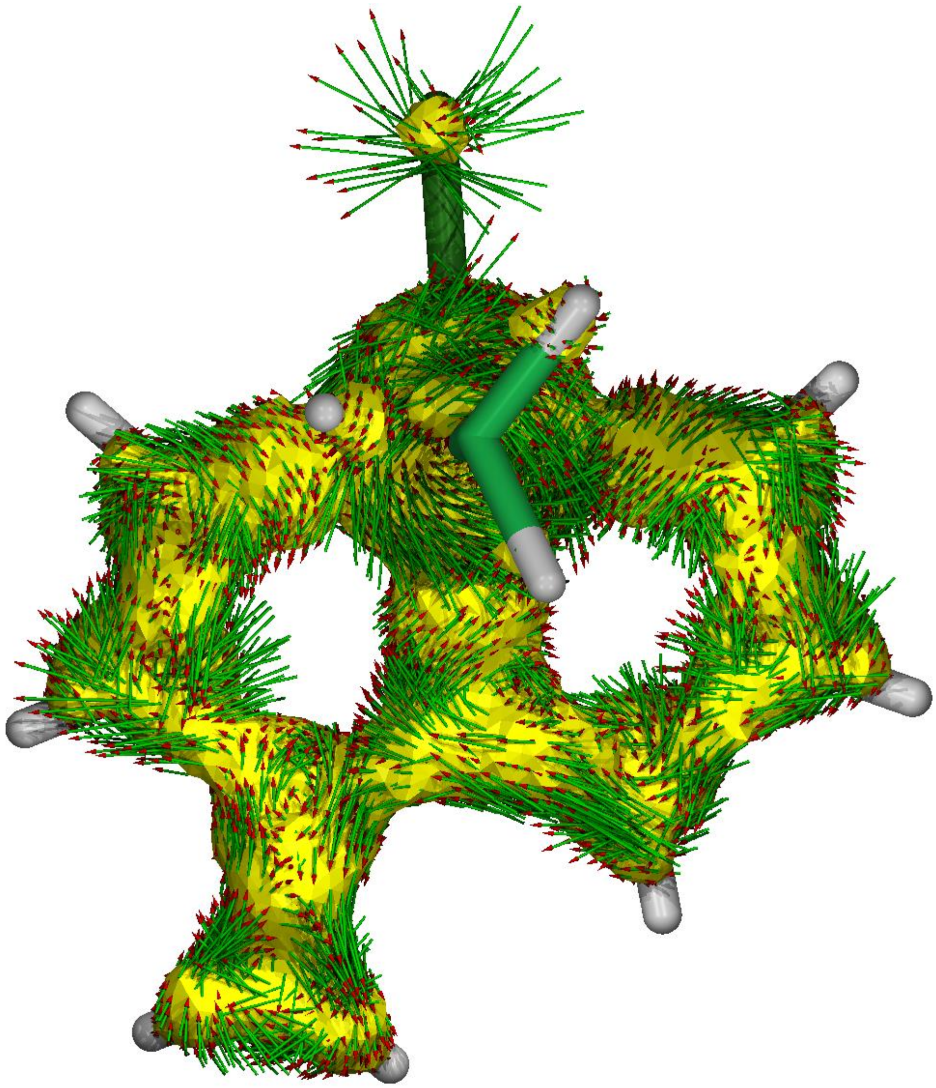

Figure S16. ACID plot of model complex **7′** from π contributions with an isosurface value of 0.025. No clear diatropic or paratropic ring current could be found in the metallacycles, related to Scheme 4.

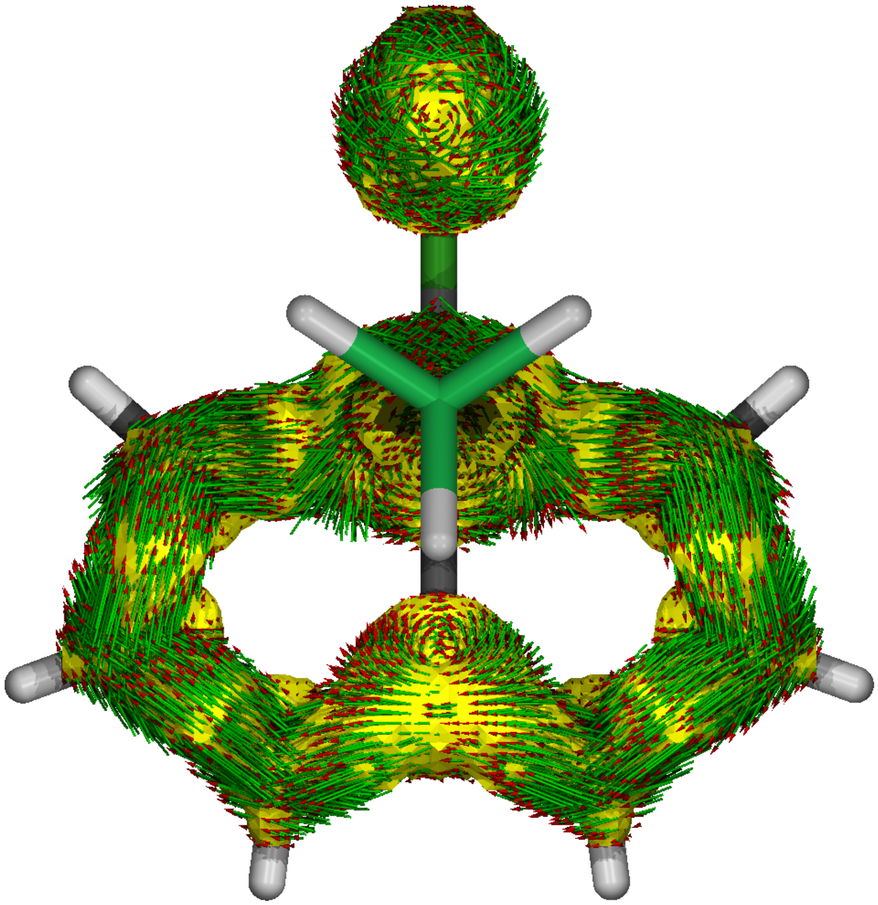

Figure S17. ACID plot of model complex **2′** from π contributions with an isosurface value of 0.025. The magnetic field vector is orthogonal to the ring plane and points upward (aromatic species exhibit clockwise diatropic circulations), related to Scheme 3C.

NMR Spectra

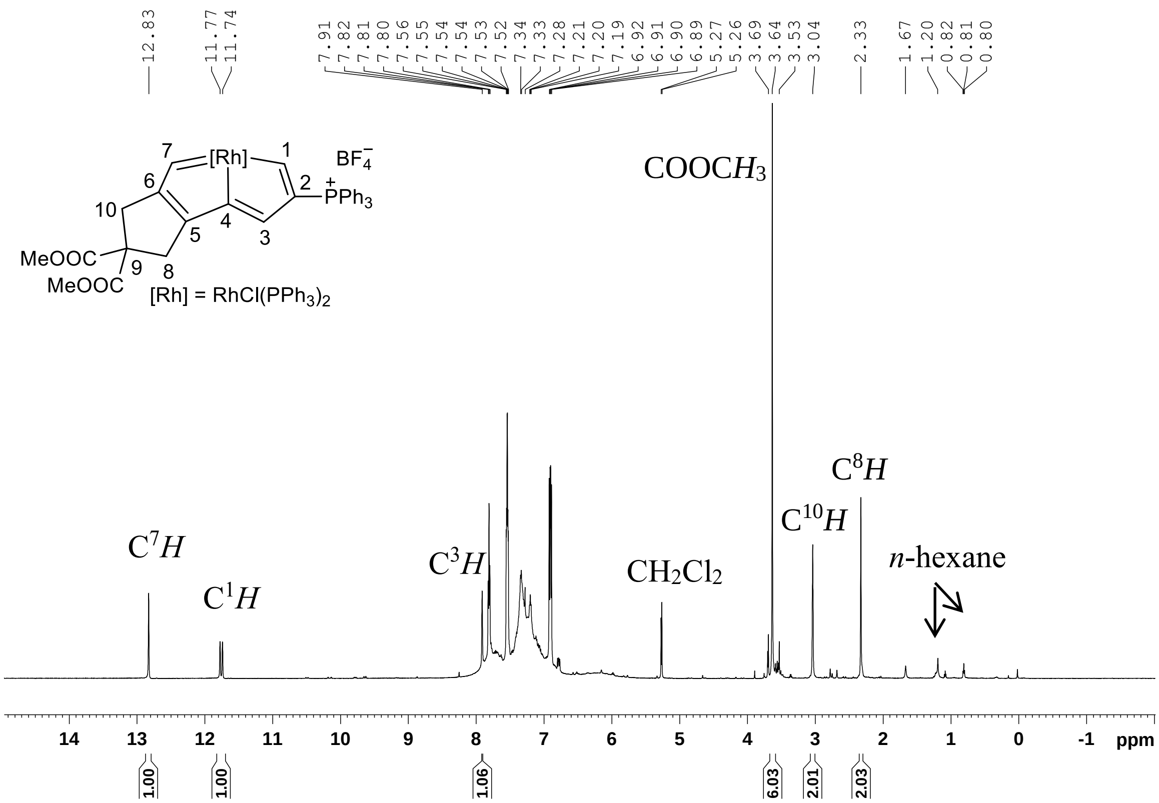

Figure S18. ^{1}H NMR spectrum (600.1 MHz) of complex **2a** in CD_2Cl_2 at room temperature, related to Scheme 2.

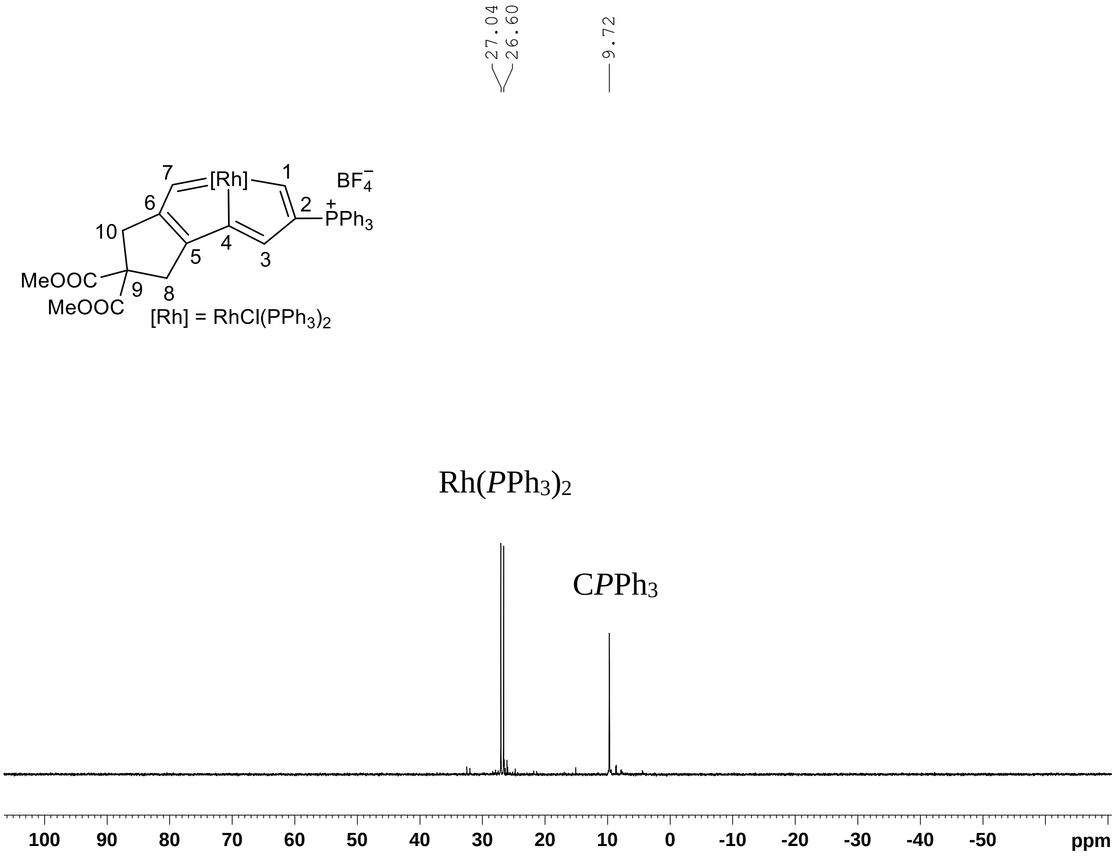

Figure S19. ^{31}P NMR spectrum (242.9 MHz) of complex **2a** in CD_2Cl_2 at room temperature, related to Scheme 2.

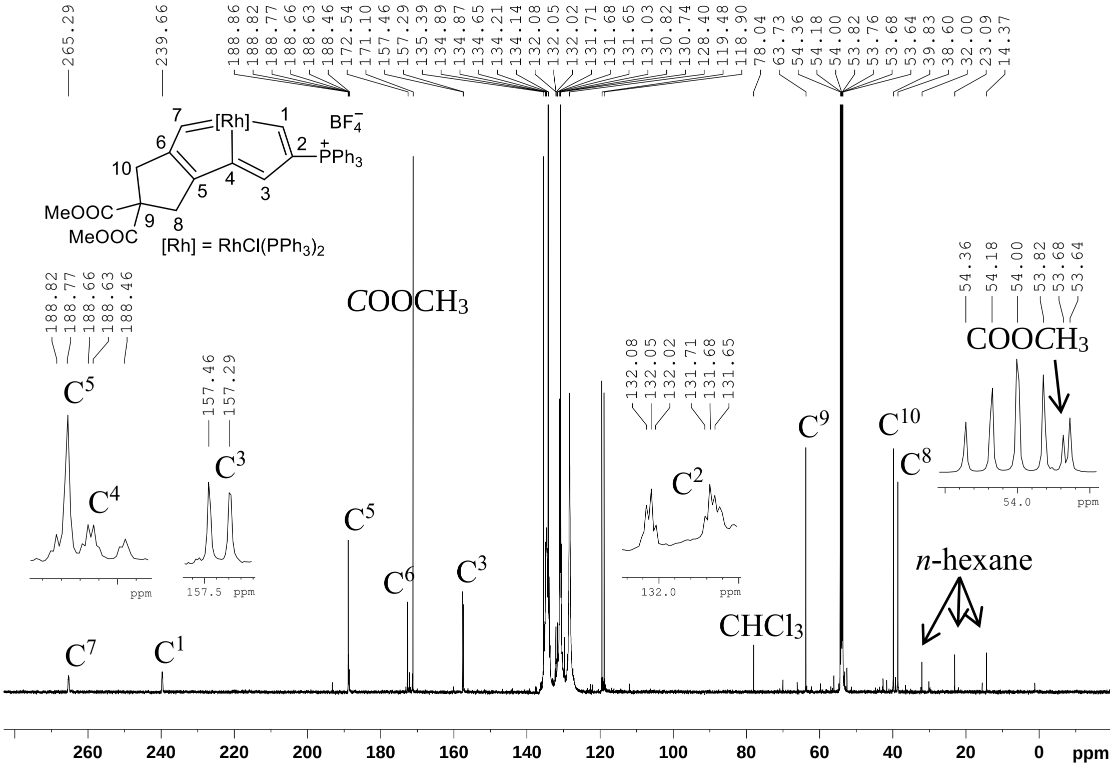

Figure S20. ^{13}C NMR spectrum (150.9 MHz) of complex **2a** in CD_2Cl_2 at room temperature, related to Scheme 2.

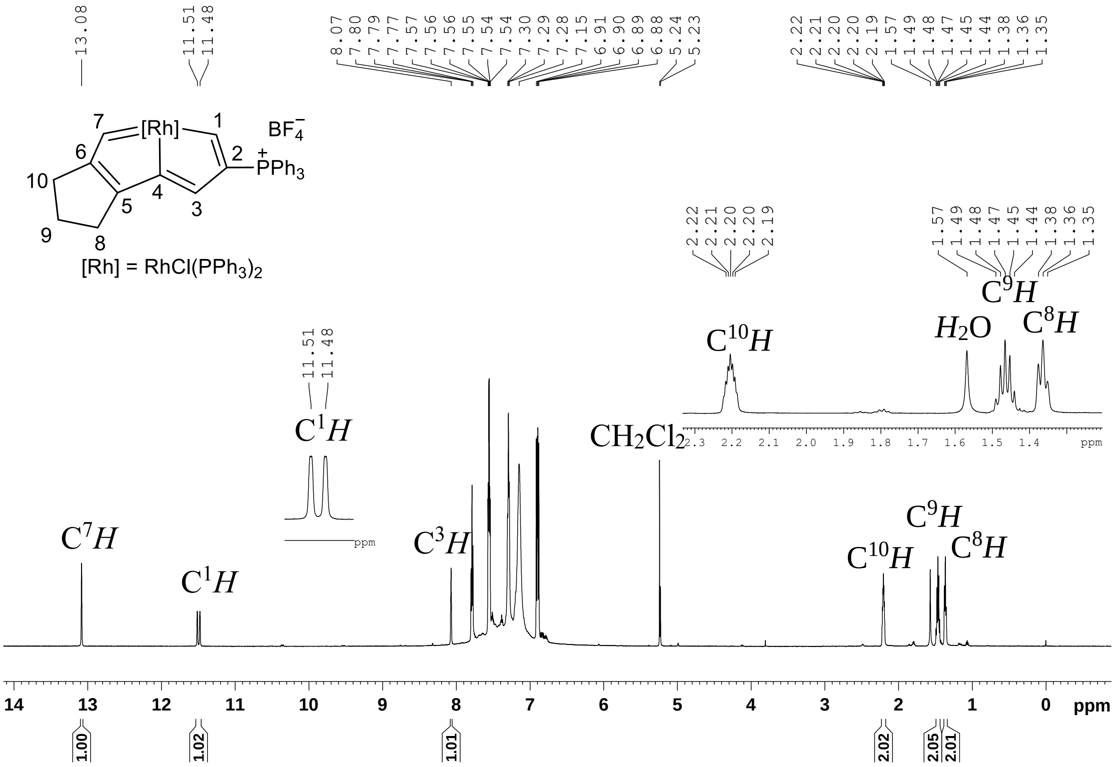

Figure S21. ^{1}H NMR spectrum (600.1 MHz) of complex **2b** in CD_2Cl_2 at room temperature, related to Scheme 2.

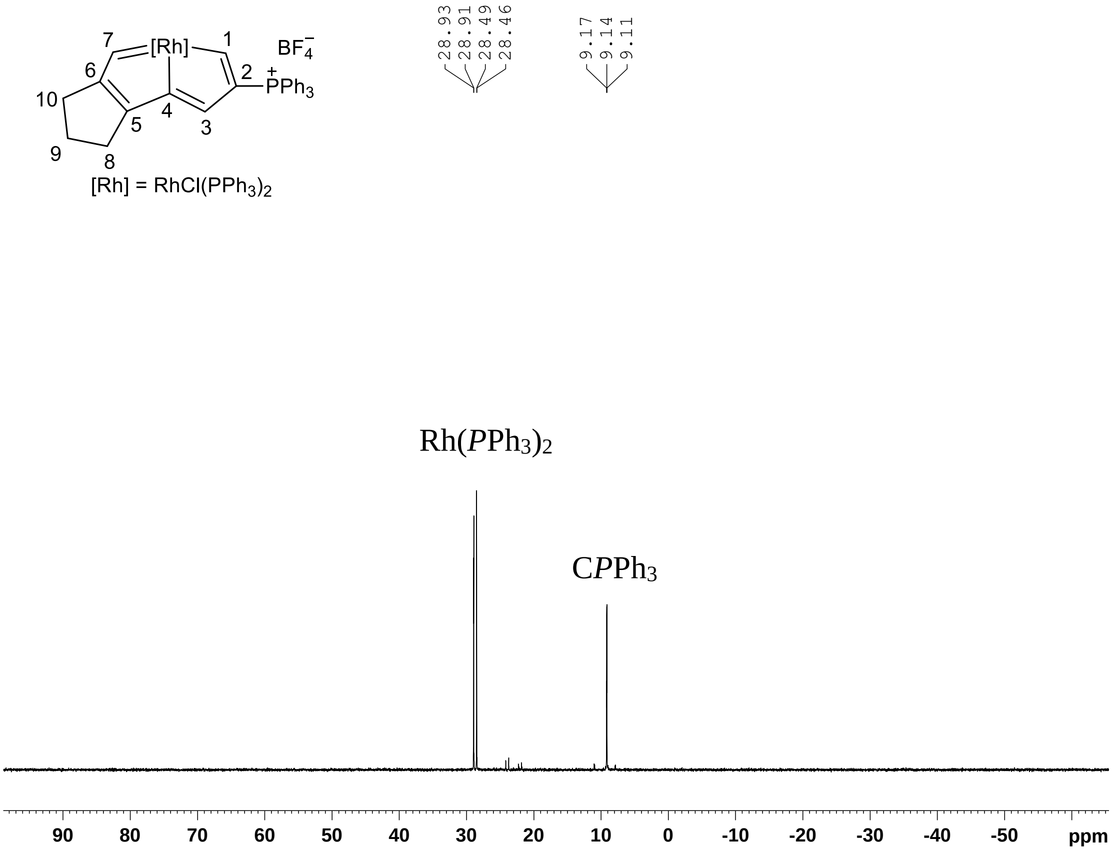

Figure S22. ^{31}P NMR spectrum (242.9 MHz) of complex **2b** in CD_2Cl_2 at room temperature, related to Scheme 2.

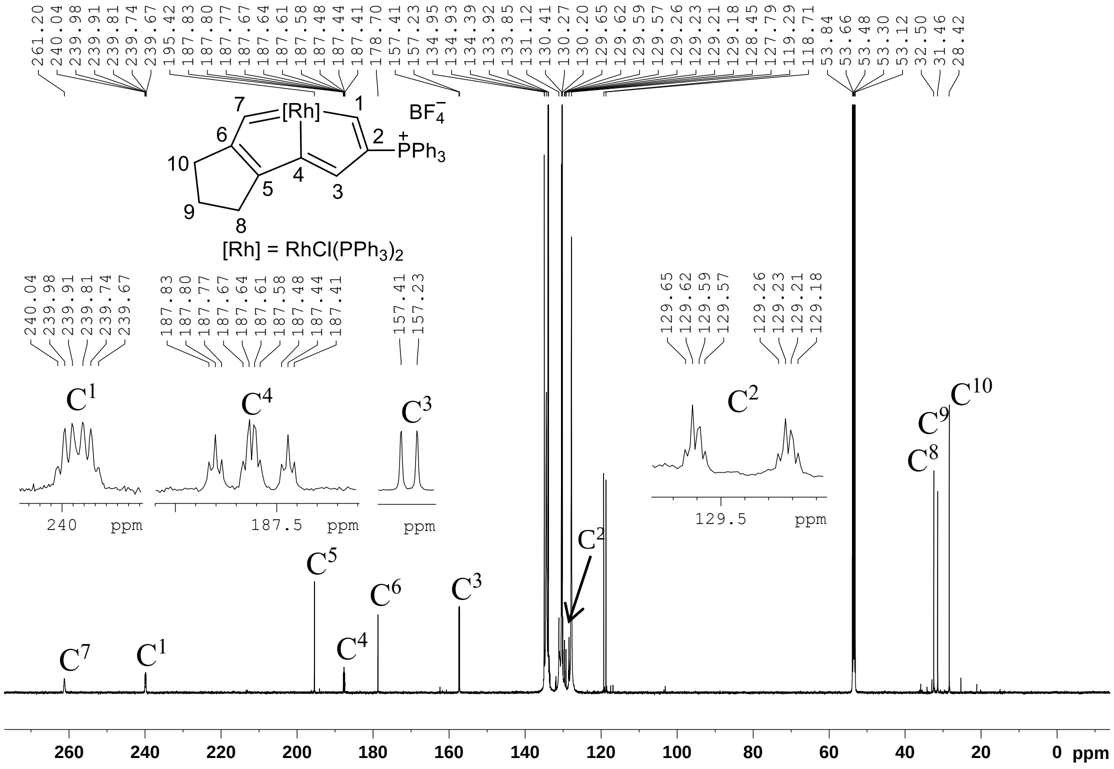

Figure S23. ^{13}C NMR spectrum (150.9 MHz) of complex **2b** in CD_2Cl_2 at room temperature, related to Scheme 2.

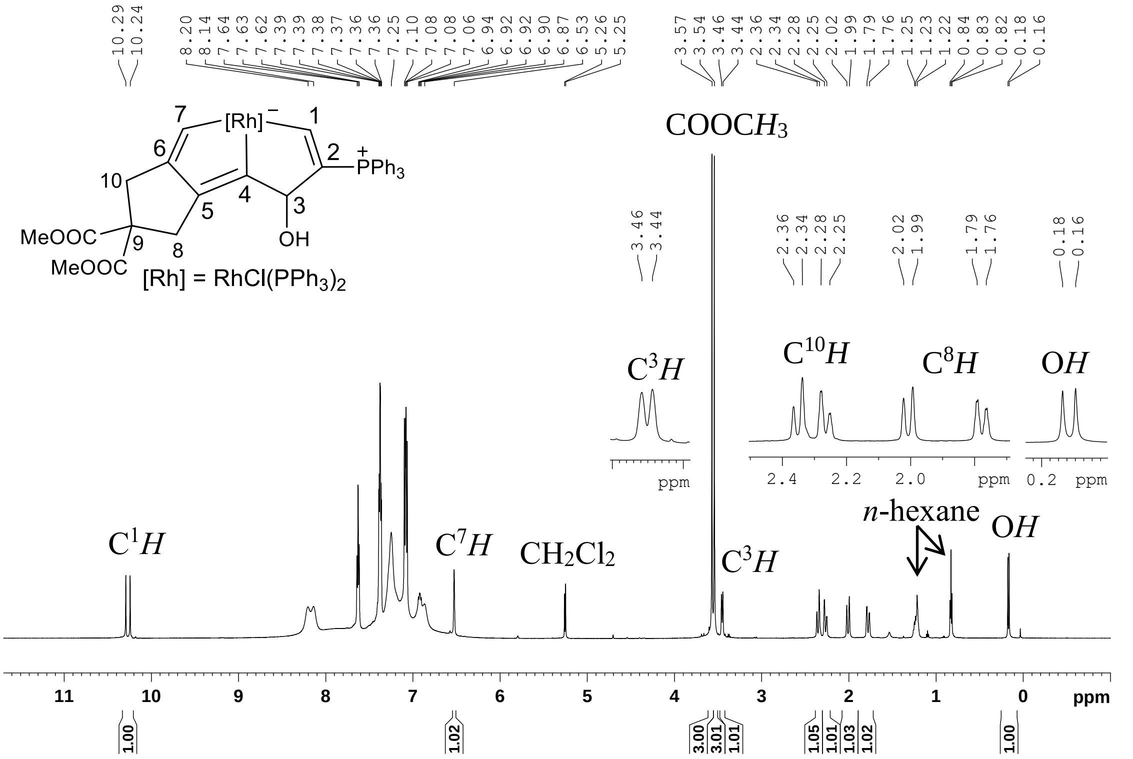

Figure S24. ^{1}H NMR spectrum (600.1 MHz) of complex **3** in CD_2Cl_2 at room temperature, related to Scheme 2.

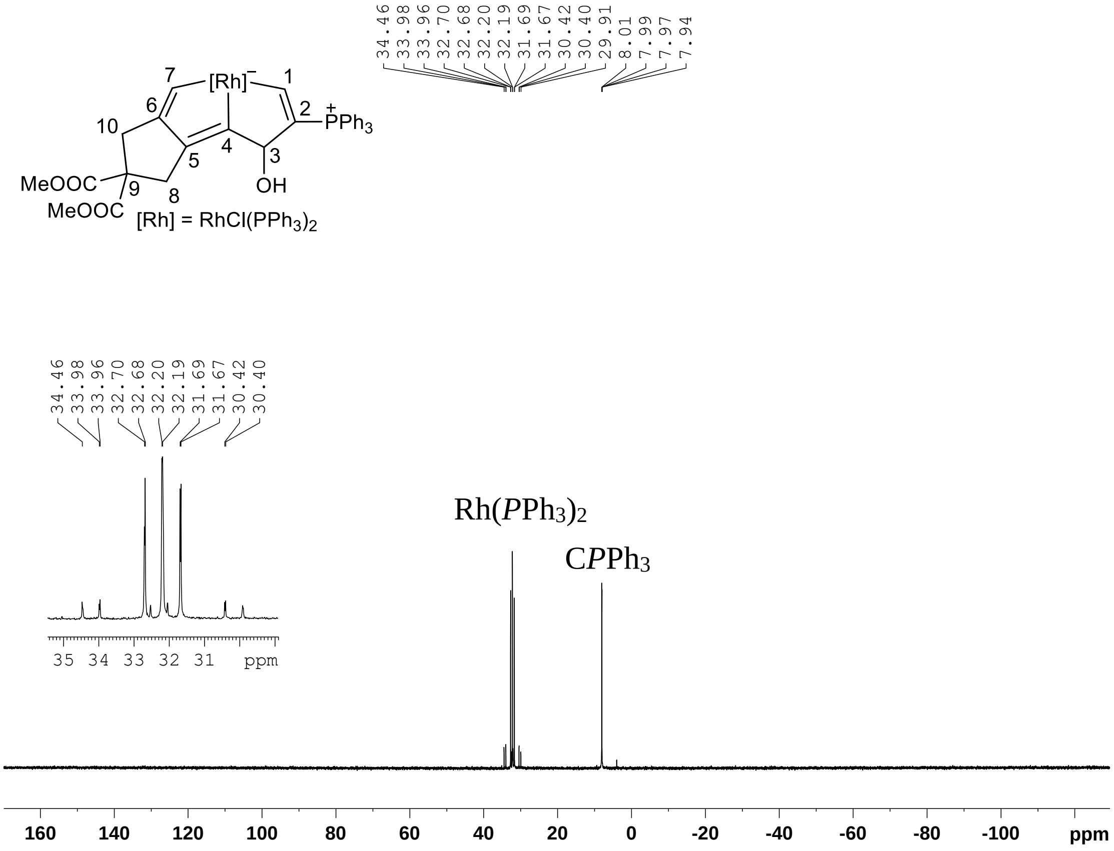

Figure S25. ^{31}P NMR spectrum (242.9 MHz) of complex **3** in CD_2Cl_2 at room temperature, related to Scheme 2.

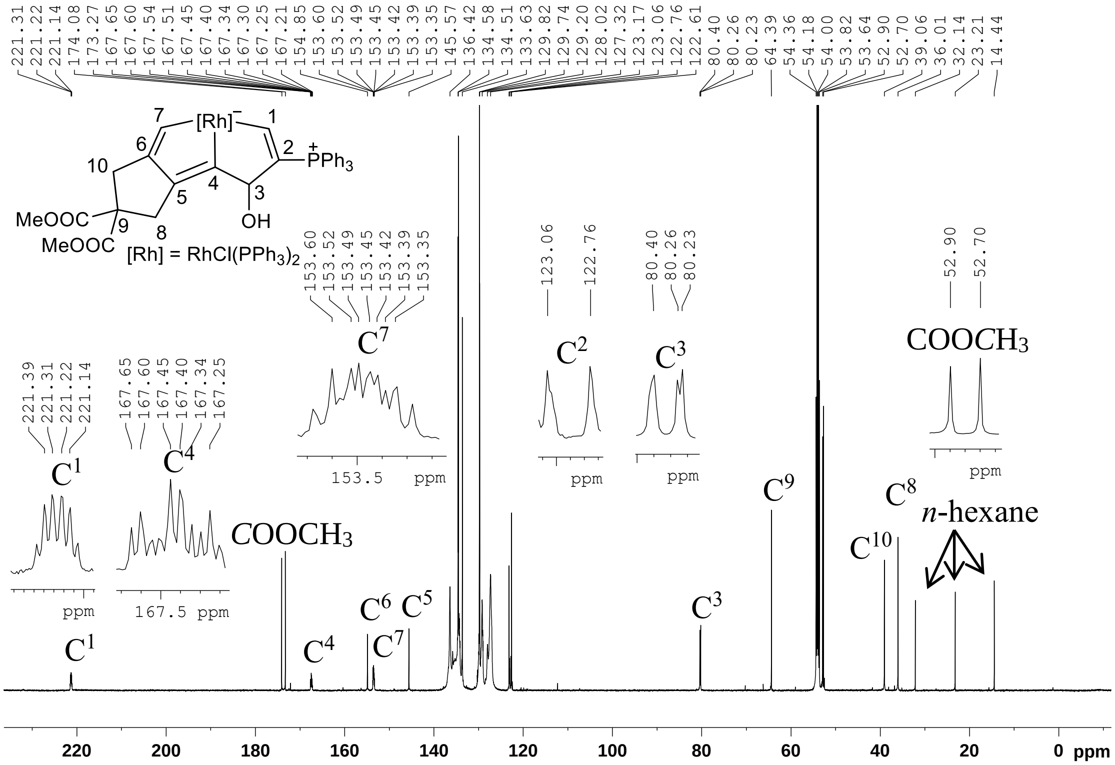

Figure S26. ^{13}C NMR spectrum (150.9 MHz) of complex **3** in CD_2Cl_2 at room temperature, related to Scheme 2.

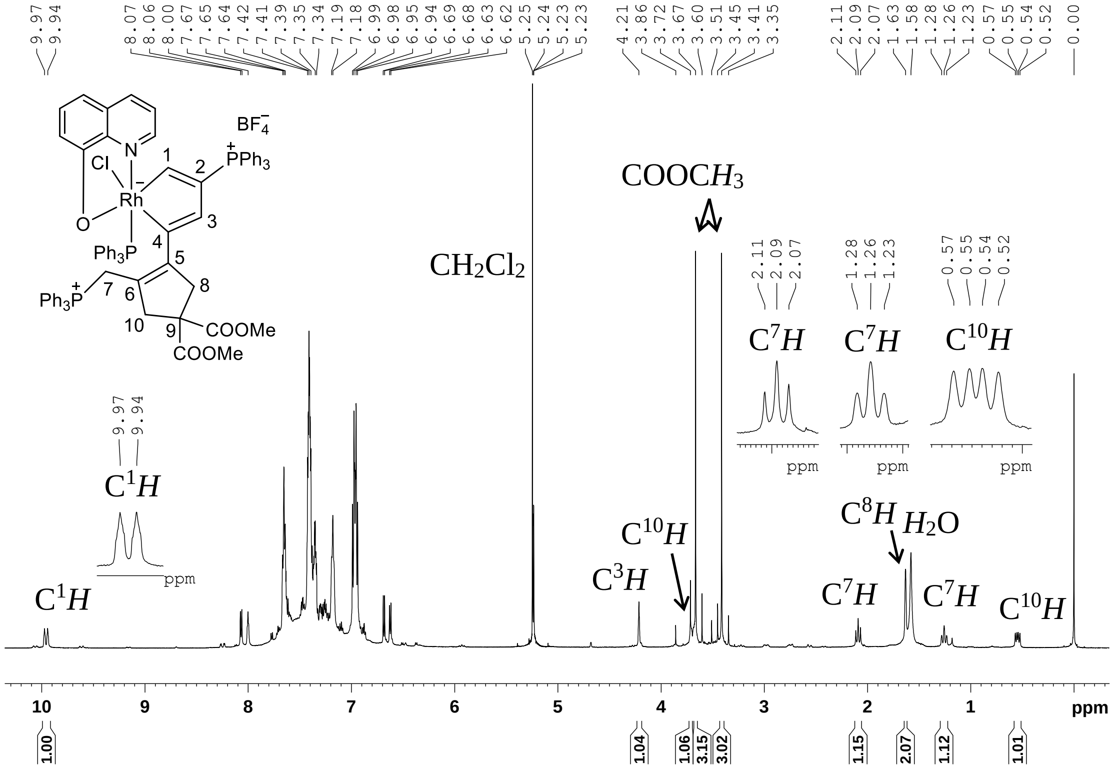

Figure S27. ^{1}H NMR spectrum (600.1 MHz) of complex **4** in CD_2Cl_2 at room temperature, related to Scheme 4A.

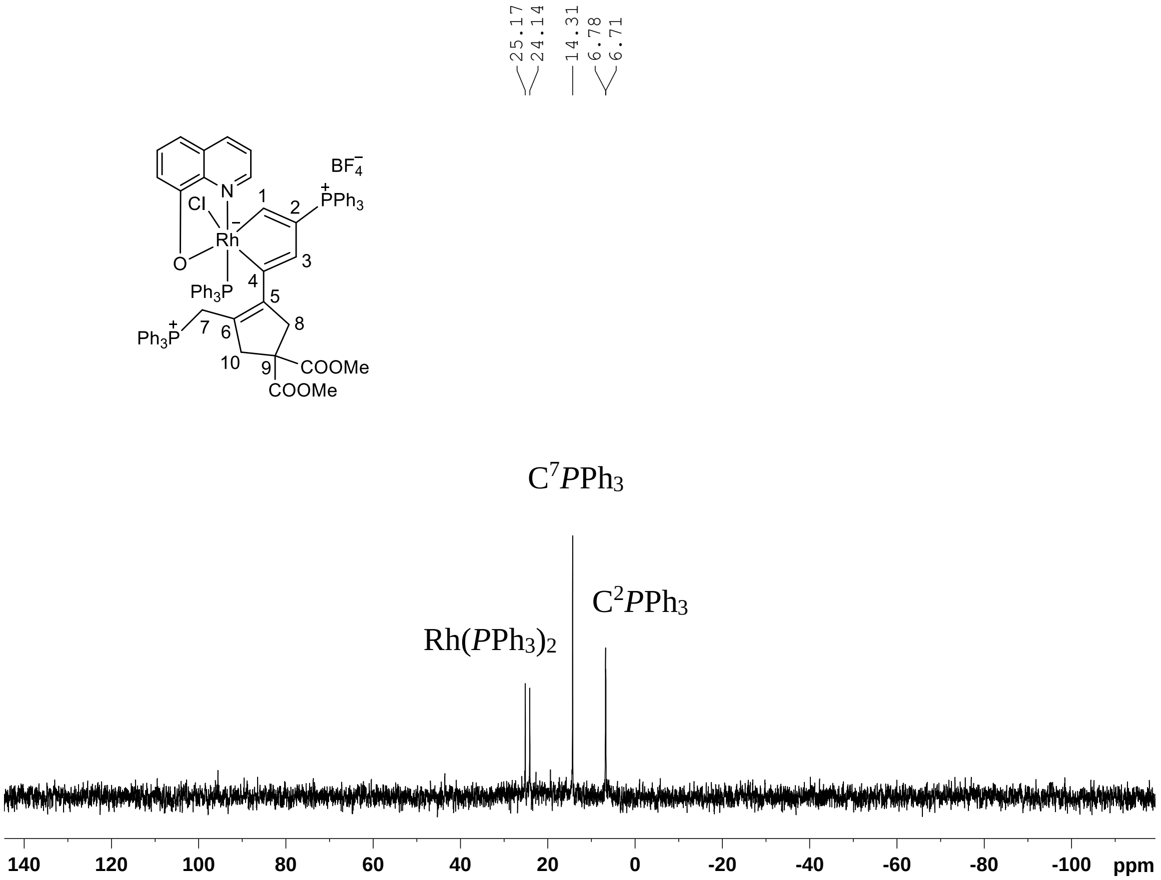

Figure S28. ^{31}P NMR spectrum (161.9 MHz) of complex **4** in CD_2Cl_2 at room temperature, related to Scheme 4A.

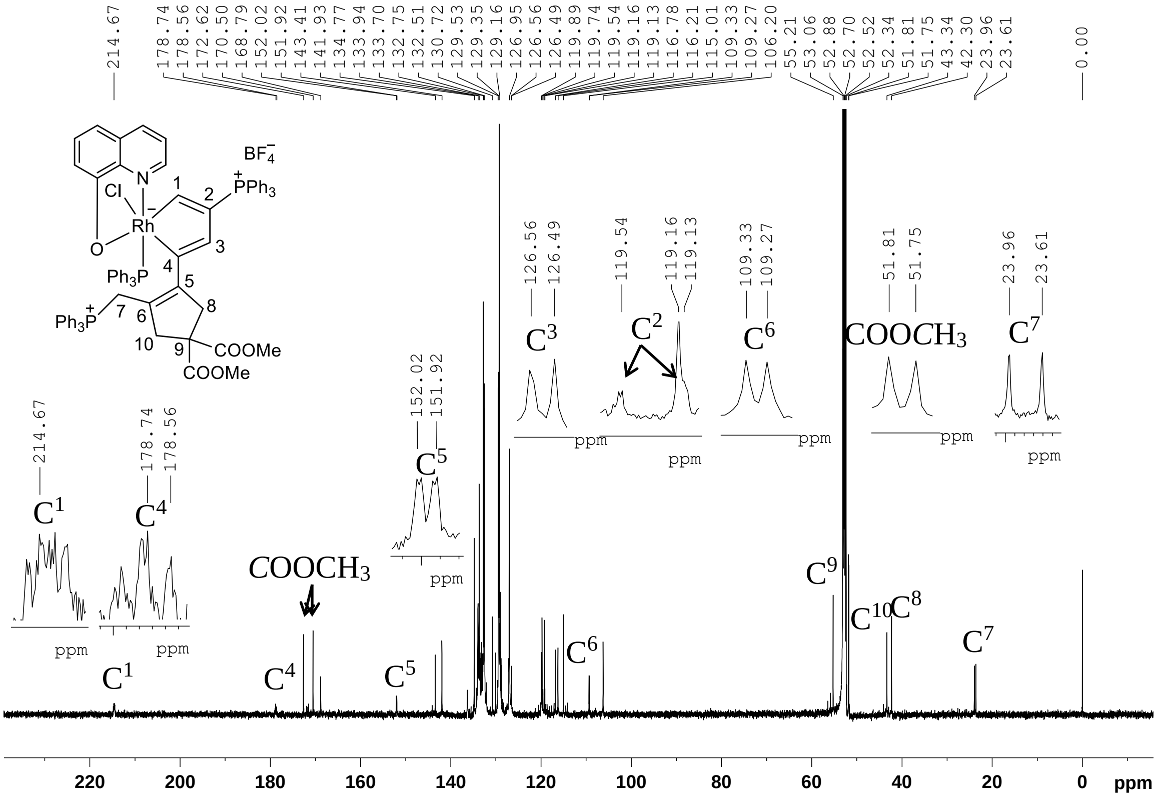

Figure S29. ^{13}C NMR spectrum (150.9 MHz) of complex **4** in CD_2Cl_2 at room temperature, related to Scheme 4A.

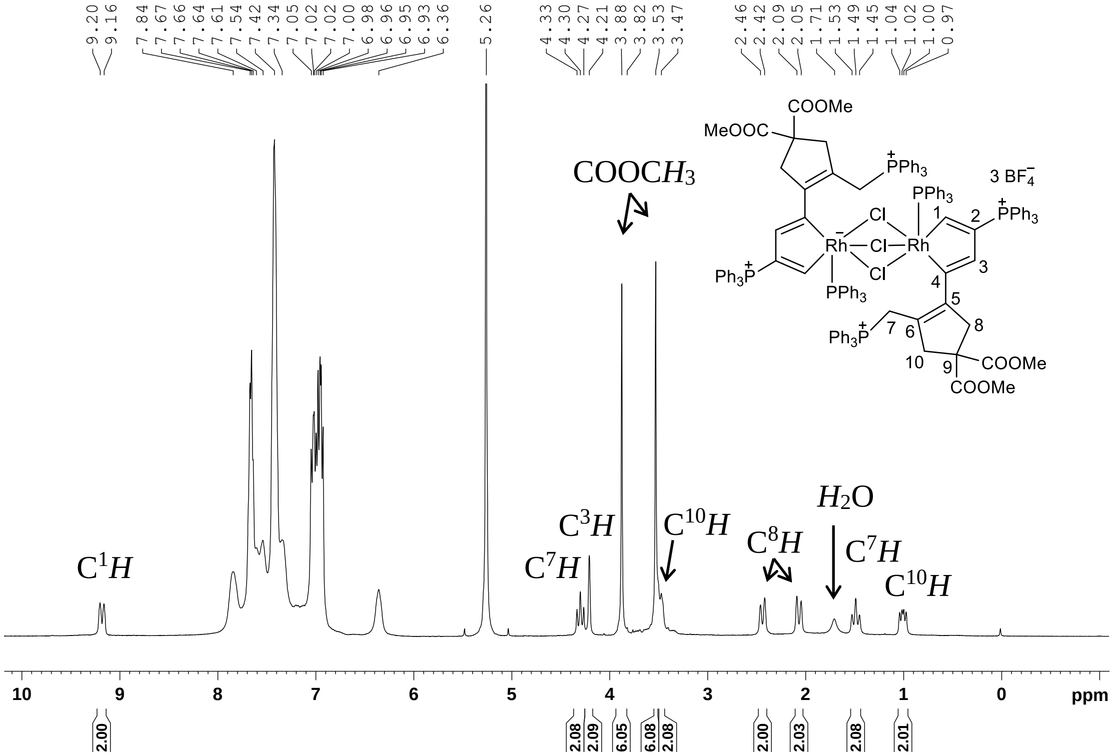

Figure S30. ^{1}H NMR spectrum (400.1 MHz) of complex **5** in CD_2Cl_2 at room temperature, related to Scheme 4A.

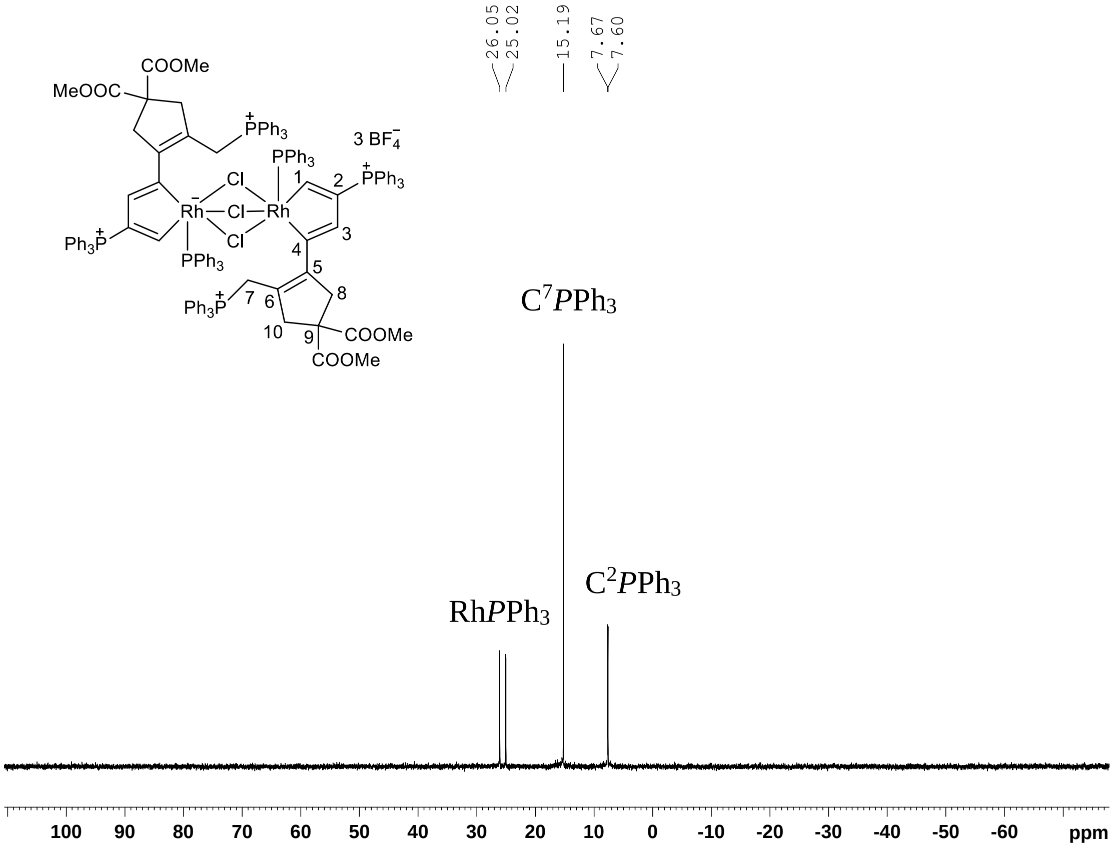

Figure S31. ^{31}P NMR spectrum (161.9 MHz) of complex **5** in CD_2Cl_2 at room temperature, related to Scheme 4A.

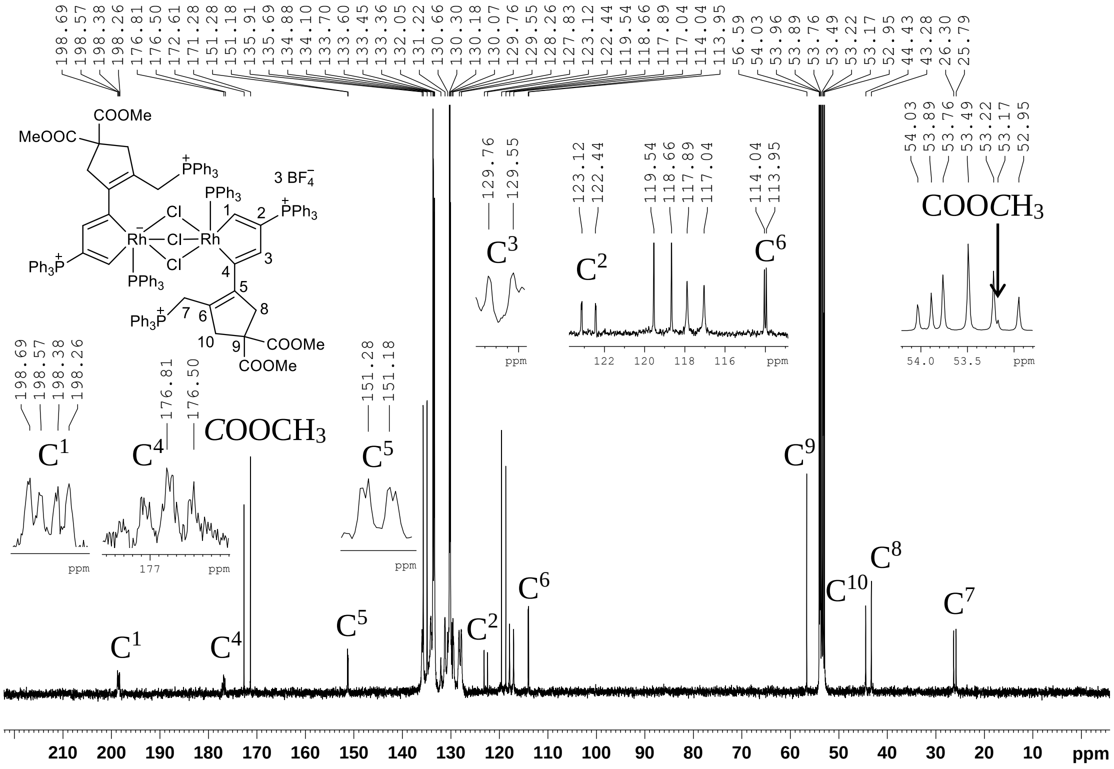

Figure S32. ^{13}C NMR spectrum (150.9 MHz) of complex **5** in CD_2Cl_2 at room temperature, related to Scheme 4A.

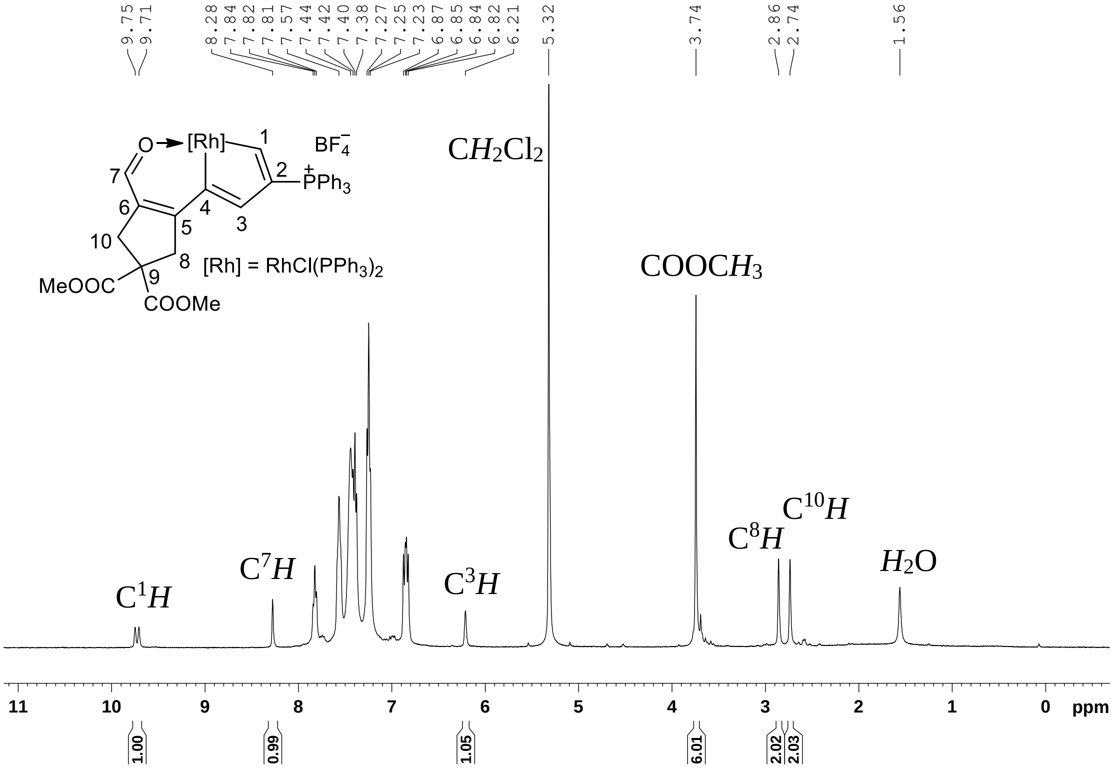

Figure S33. ^{1}H NMR spectrum (400.1 MHz) of complex **6** in CD_2Cl_2 at room temperature, related to Scheme 4A.

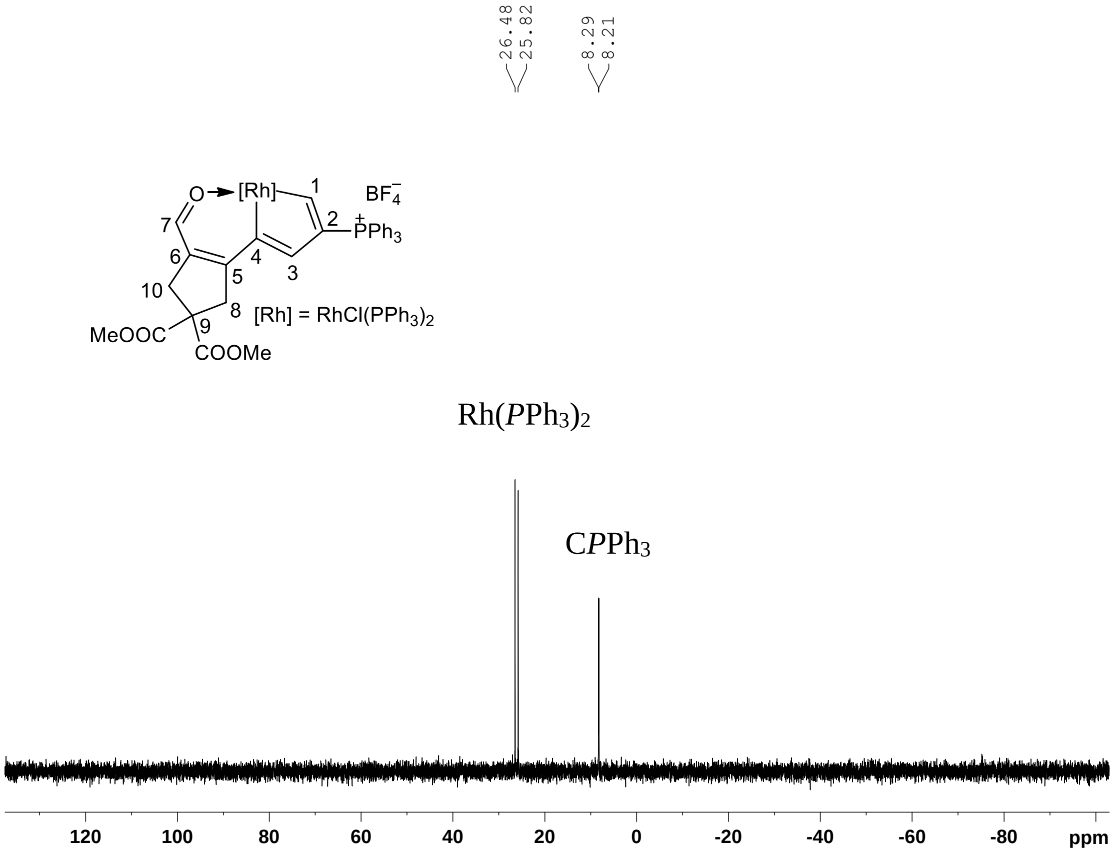

Figure S34. ^{31}P NMR spectrum (161.9 MHz) of complex **6** in CD_2Cl_2 at room temperature, related to Scheme 4A.

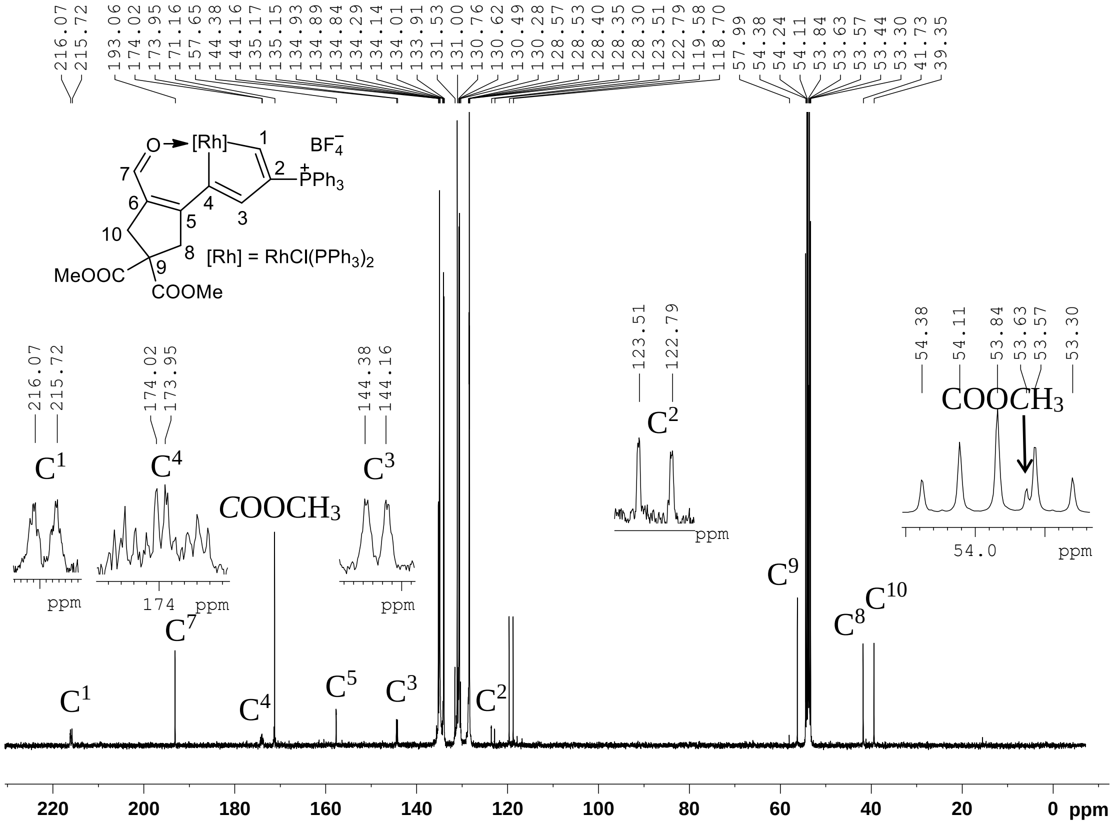

Figure S35. ^{13}C NMR spectrum (100.6 MHz) of complex **6** in CD_2Cl_2 at room temperature, related to Scheme 4A.

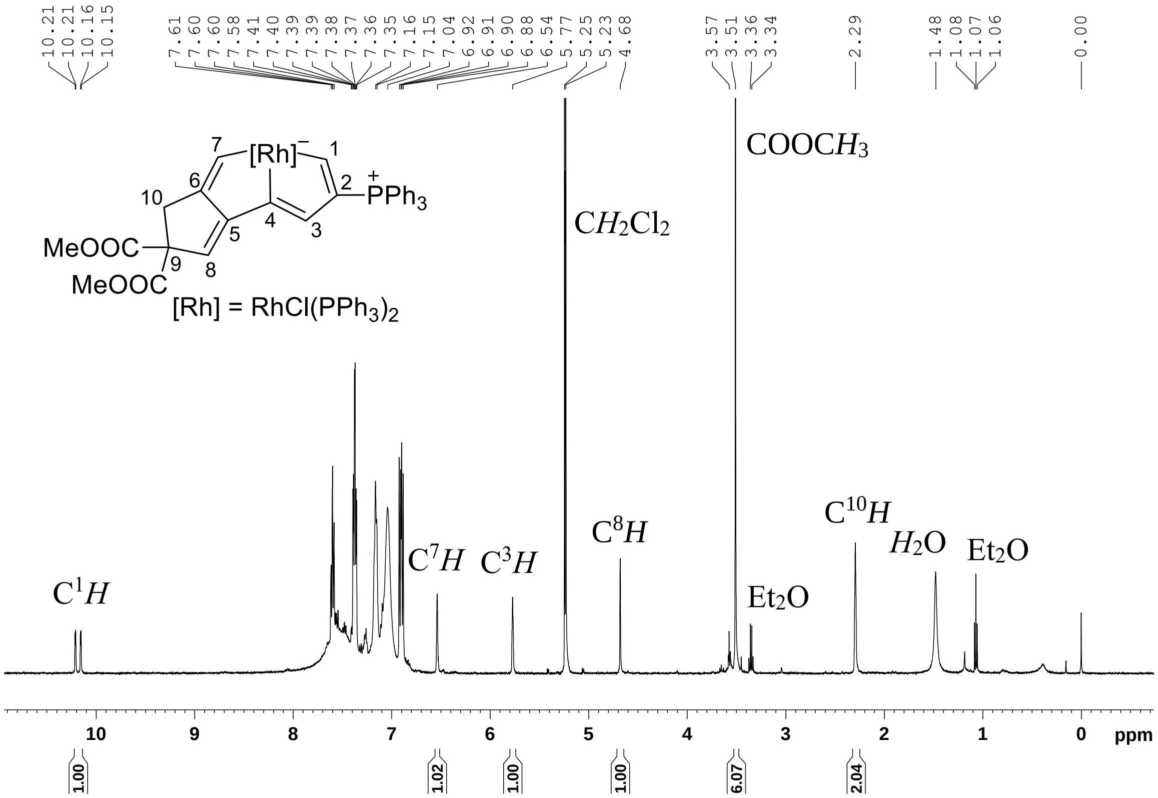

Figure S36. ^{1}H NMR spectrum (500.2 MHz) of complex **7** in CD_2Cl_2 at room temperature, related to Scheme 4A.

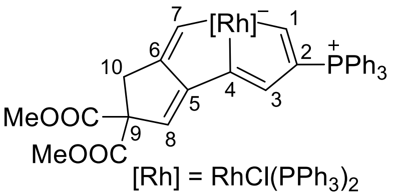

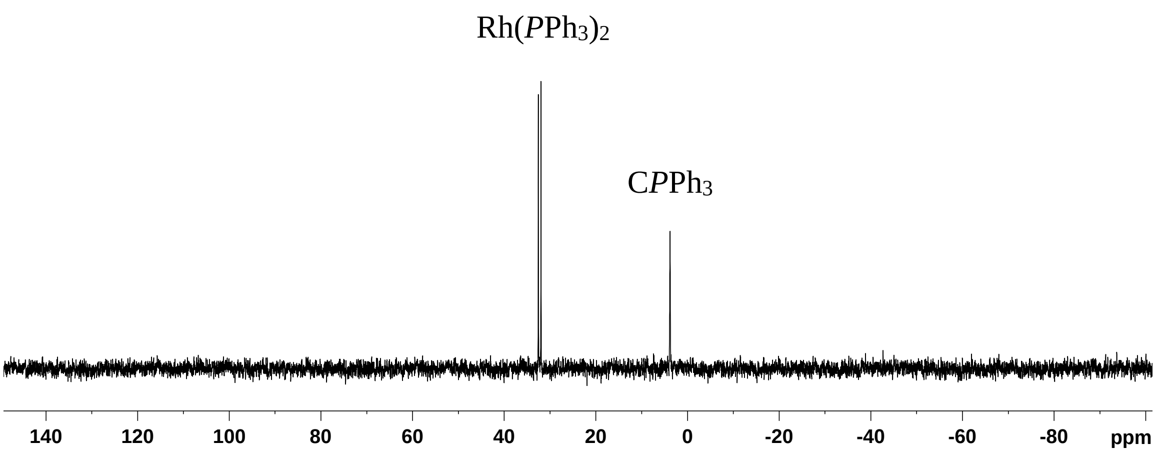

Figure S37. ^{31}P NMR spectrum (202.5 MHz) of complex **7** in CD_2Cl_2 at room temperature, related to Scheme 4A.

Table S1. Crystal data and structure refinement of 2a, 3 and 4, related to Schemes 2 and 4.

	**2a**·1.5$C_2H_4Cl_2$	**3**·C_4H_8O	**4**·1.5CH_2Cl_2
Empirical formula	$C_{71}H_{64}BCl_4F_4O_4P_3Rh$	$C_{72}H_{67}ClO_6RhP_3$	$C_{78.5}H_{68}BCl_4F_4NO_5P_3Rh$
Mol. weight	1405.65	1259.52	1529.77
Temperature [K]	153.00(14)	168(20)	100.01(10)
Crystal system	Monoclinic	Monoclinic	Triclinic
Space group	$P2_1/n$	P2(1)/c	P-1
a [Å]	12.27280(13)	18.5774(4)	10.4683(4)
b [Å]	20.6749(3)	13.0396(3)	13.4616(4)
c [Å]	25.2565(3)	27.1373(5)	25.3697(9)
α [°]	90	90	94.693(3)
β [°]	98.6364(11)	99.351(2)	95.913(3)
γ [°]	90	90	97.674(3)
V [$Å^3$]	6335.89(13)	6486.4(2)	3507.6(2)
Z	4	4	2
ρ_{calcd} [g cm^{-3}]	1.474	1.290	1.448
μ [mm^{-1}]	4.961	0.429	4.546
F(000)	2884.0	2616.0	1570.0
Crystal size [mm^3]	0.2 × 0.2 × 0.1	0.20×0.20×0.20	0.1 × 0.1 × 0.05
Radiation	CuKα (λ = 1.54184)	MoKα (λ = 0.71073)	CuKα (λ = 1.54184)
2θ range [°]	7.608 to 124.592	3.474 to 49.998	7.04 to 134.996
Coll. refl.	35912	31595	24419
Indep. refl.	9985	11408	12635
data/restraints/params	9985/1/822	11408/150/825	12635/12/1090
GOF on F^2	1.046	1.099	1.027
R_1/wR_2 [$I \geq 2\sigma(I)$]	0.0433/0.1120	0.0572/0.1556	0.0539/0.1438
R_1/wR_2 (all data)	0.0482/0.1172	0.0694/0.1631	0.0576/0.1484
Largest peak/hole [e $Å^{-3}$]	1.45/-1.04	1.90/-0.76	1.93/-1.44

Table S2. Crystal data and structure refinement of 5, 6 and 7, related to Scheme 4.

	**5**·$6CH_2Cl_2$	**6**·$1.5CH_2Cl_2$	**7**·$1.25CH_2Cl_2$
Empirical formula	$C_{142}H_{130}B_3Cl_{15}F_{12}O_8P_6Rh_2$	$C_{69.5}H_{61}BCl_4F_4O_5P_3Rh$	$C_{69.25}H_{59.5}Cl_{3.5}O_4P_3Rh$
Mol. weight	3148.27	1400.61	1275.56
Temperature [K]	173(2)	179(9)	178(5)
Crystal system	Triclinic	Monoclinic	Monoclinic
Space group	P-1	$P2_1/n$	$P2_1/c$
a [Å]	14.4220(4)	12.3594(2)	12.79757(18)
b [Å]	15.2205(4)	20.3965(4)	21.9384(3)
c [Å]	33.0221(7)	25.3431(5)	22.2894(3)
α [°]	94.6105(18)	90	90
β [°]	91.6756(19)	97.6116(19)	101.1559(15)
γ [°]	92.942(2)	90	90
V [Å^3]	7211.6(3)	6332.4(2)	6139.70(15)
Z	2	4	4
ρ_{calcd} [g cm^{-3}]	1.450	1.469	1.380
μ [mm^{-1}]	0.644	4.974	4.773
F(000)	3208.0	2868.0	2626.0
Crystal size [mm]	0.4 × 0.2 × 0.2	0.2 × 0.15 × 0.1	0.2 × 0.2 × 0.2
Radiation	MoKα (λ = 0.71073)	CuKα (λ = 1.54184)	CuKα (λ = 1.54184)
2*θ* range [°]	3.05 to 50	7.038 to 124.488	7.04 to 124.286
Coll. refl.	48907	21698	20543
Indep. refl.	25391	9907	9589
data/restraints/params	25391/73/1787	9907/18/831	9589/48/790
GOF on F^2	1.063	1.065	1.046
R_1/wR_2 [$I \geq 2\sigma(I)$]	0.0675 / 0.1682	0.0496/0.1225	0.0399/0.1176
R_1/wR_2 (all data)	0.0894 / 0.1801	0.0566/0.1283	0.0451/0.1228
Largest peak/hole [e Å^{-3}]	1.71/-0.91	1.07/-1.17	1.38/-0.69

Table S3. Wiberg bond indices of model metallapentalenes Os-1, Os-2, Ru-1, Ru-2 and 2′, related to Figure 3.

Compound	M–C1/ M–C7	M–C4	M–Cl/ M–CO	C1–C2/ C6–C7	C2–C3/ C5–C6	C3–C4/ C4–C5
**Os-1**	1.02	0.85	0.62	1.48	1.39	1.36
**Os-2**	0.96	0.72	1.30	1.51	1.36	1.41
**Ru-1**	0.91	0.77	0.73	1.50	1.38	1.38
**Ru-2**	0.86	0.69	1.11	1.54	1.35	1.41
**2′**	0.76	0.74	0.60	1.56	1.33	1.41

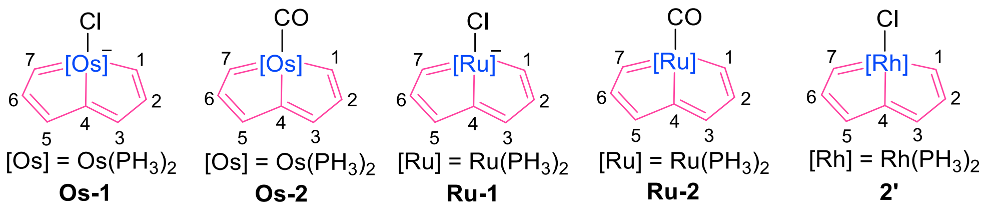

Transparent Methods

General Information. All syntheses were performed under an N_2 atmosphere using standard Schlenk techniques unless otherwise stated. Hexane, tetrahydrofuran and diethyl ether solvents were distilled from sodium/benzophenone. Dichloromethane was distilled over calcium hydride under N_2 prior to use. Triynes **1a** and **1b** (purchased from J&K® Chemical) and other reagents were used as received from commercial sources without further purification. Column chromatography was performed on silica gel (200-300 mesh) in air. Nuclear magnetic resonance (NMR) spectroscopy was performed using a Bruker Advance II 400 spectrometer, a Bruker Advance III 500 spectrometer or a Bruker Ascend III 600 spectrometer at room temperature. The ^{1}H and ^{13}C NMR chemical shifts (δ) are relative to tetramethylsilane, and the ^{31}P NMR chemical shifts are relative to 85% H_3PO_4. The absolute values of the coupling constants are given in hertz (Hz). Elemental analyses were performed on a Vario EL III elemental analyzer. Thermal gravimetric analyses (TGA) were carried out on an SDT Q600 at a heating rate of 10 °C min^{-1} from room temperature to 800 °C under an air atmosphere.

Single-Crystal X-Ray Diffraction Experiments. Single-crystal X-ray diffraction data were collected on an Oxford Gemini S Ultra CCD area detector with graphite-monochromated Mo Kα radiation (λ = 0.71073 Å) for **3** and **5** and mirror-monochromated Cu Kα radiation (λ = 1.54184 Å) for **2a**, **6** and **7**. An Agilent SuperNova Dual system with mirror-monochromated Cu Kα radiation (λ = 1.54184 Å) was used for **4**. Absorption corrections were applied by using the program CrysAlis (Version 1.171.36.24 for **2a**, **6** and **7**, Version 1.171.39.46e for **3** and **5**, and Version 1.171.37.35 for **4**; multi-scan mode). Using Olex2 (Dolomanov et al., 2009), the structures were solved using the ShelXT (Sheldrick, 2015b) structure solution program using the intrinsic phasing method (**2a**, **3**, **4**, **5**, **6** and **7**), and all of the structures were refined with the ShelXL (Sheldrick, 2015a) refinement package using least-squares minimization. Non-H atoms were refined anisotropically unless otherwise stated. Hydrogen atoms were introduced at their geometric positions and refined as riding atoms unless otherwise stated. Single crystals suitable for X-ray diffraction were grown from a solution of $ClCH_2CH_2Cl$ (**2a**), THF (**3**), or CH_2Cl_2 (**4**, **5**, **6** and **7**) layered with hexane. The THF solvent molecules in **3**, some of the CH_2Cl_2 solvent molecules in **5**, **6** and **7**, and two of the BF_4 counter anions in **5** were disordered and refined with suitable constraints. CCDC-1506335 (**2a**), CCDC-1506333 (**3**), CCDC-1848362 (**4**), CCDC-1848363 (**5**), CCDC-1848364 (**6**) and CCDC-1848365 (**7**) contain the supplementary crystallographic data for this paper. Further details on the crystal data, data collection, and refinements are provided in Tables S1 and S2. These data can be obtained free of charge from the Cambridge Crystallographic Data Centre via www.ccdc.cam.ac.uk/data_request/cif.

Computational details. All structures were optimized at the B3LYP level of DFT (Becke, 1993; Lee et al., 1988; Miehlich et al., 1989). Frequency calculations were performed to identify all the stationary points as minima (zero imaginary frequency). In the B3LYP calculations, the effective core potentials (ECPs) of Hay and Wadt with a double-ζ valence basis set (LanL2DZ) (Hay and Wadt, 1985) were used to describe the Rh, Os, Ru, Cl, and P atoms, whereas the standard 6-311++G** basis set was used for the C, O and H atoms. Polarization functions were added for Rh (ζ(f) = 1.350), Os (ζ(f) = 0.886), Ru (ζ(f) = 1.235), Cl (ζ(d) = 0.514) and P (ζ(d) = 0.340) (Huzinaga, 1984) in all calculations. All optimizations were performed with the Gaussian 09 software package (Frisch et al., 2013), whereas the Wiberg bond index (Wiberg, 1968) calculations were carried out with the NBO 6.0 program (Glendening, 2013) interfaced with the Gaussian 09 program. Nucleus-independent chemical shift (NICS) (Chen et al. 2005; Fallah-Bagher-Shaidaei et al., 2006; Schleyer et al., 1996) values were calculated at the same level. The anisotropy of the induced current density (ACID) calculations was carried out with the ACID program (Geuenich et al. 2005). The energies (in kcal/mol) are given and include the zero-point energy corrections.

Experimental Procedures

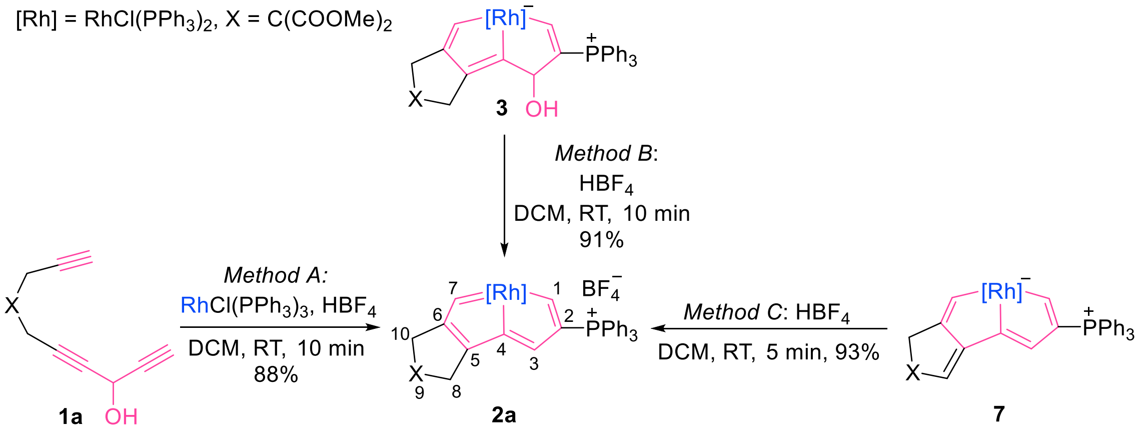

Synthesis and characterization of complex 2a: *Method A*: A dichloromethane solution (5 mL) of **1a** (1.03 g, 3.93 mmol) and a solution of HBF_4 (48 wt% solution in H_2O, 1.20 mL, 9.25 mmol) were sequentially added to a red solution of $RhCl(PPh_3)_3$ (3.04 g, 3.29 mmol) in dichloromethane (120 mL) slowly. The reaction mixture was stirred at room temperature for 10 min to yield a red solution. The solvent volume was reduced to approximately 15 mL under vacuum, and the mixture was purified by column chromatography on silica gel (eluent: dichloromethane/acetone = 20:1) to afford complex **2a** as a red solid. Yield: 3.63 g, 88%. *Method B*: HBF_4 (48 wt% solution in H_2O, 0.24 mL, 1.9 mmol) was added to a suspension of complex **3** (800 mg, 0.67 mmol) in dichloromethane (30 mL). The reaction mixture was stirred at room temperature for 10 min to yield a red solution. The solvent volume was reduced to approximately 5 mL under vacuum, and

the mixture was purified by column chromatography on silica gel (eluent: dichloromethane/acetone = 20:1) to afford complex **2a** as a red solid. Yield: 771 mg, 91%. *Method C*: HBF_4 (48 wt% solution in H_2O, 83 μL, 0.64 mmol) was added to a suspension of complex **7** (250 mg, 0.214 mmol) in dichloromethane (8 mL). The reaction mixture was stirred at room temperature for 5 min to yield a red solution. The solvent volume was reduced to approximately 2 mL under vacuum, and the mixture was purified by column chromatography on silica gel (eluent: dichloromethane/acetone = 20:1) to afford complex **2a** as a red solid. Yield: 250 mg, 93%. ^{1}H NMR (600.1 MHz, CD_2Cl_2): δ 12.83 (s, 1H, C^7H), 11.76 (d, J(HP) = 21.49 Hz, 1H, C^1H), 7.91 (s, 1H, C^3H), 6.89–7.82 (45H, Ph), 3.64 (s, 6H, $COOCH_3$), 3.04 (s, 2H, $C^{10}H$), 2.33 ppm (s, 2H, C^8H). ^{31}P NMR (242.9 MHz, CD_2Cl_2): δ 26.82 (d, J(PRh) = 105.77 Hz, RhPPh_3), 9.72 ppm (s, CPPh_3). ^{13}C NMR (150.9 MHz, CD_2Cl_2, plus ^{13}C DEPT-135, ^{1}H-^{13}C HSQC and ^{1}H-^{13}C HMBC): δ 265.29 (br, C^7), 239.66 (br, C^1), 188.77 (s, C^5), 188.66 (ddt, J(CRh) = 27.43 Hz, J(CP) = 25.42 Hz, J(CP) = 4.93 Hz, C^4), 172.54 (s, C^6), 171.10 (s, $COOCH_3$), 157.38 (d, J(CP) = 25.91 Hz, C^3), 134.14–135.39 (Ph), 131.86 (ddt, J(CP) = 55.99 Hz, J(CRh) = 4.31 Hz, J(CP) = 4.31 Hz, C^2), 128.40–131.03 (Ph), 119.19 (d, J(CP) = 87.55 Hz, Ph), 63.73 (s, C^9), 53.68(s, $COOCH_3$), 39.83 (s, C^8), 38.60 ppm (s, C^{10}). Anal. Calcd (%) for $C_{68}H_{58}BClF_4O_4P_3Rh$: C, 64.96; H, 4.65. Found: C, 65.04; H, 4.90.

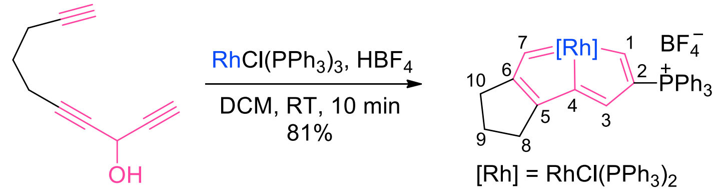

1b **2b**

Synthesis and characterization of complex 2b: 1b (180 mg, 1.23 mmol) and HBF_4 (48 wt% solution in H_2O, 0.36 mL, 2.8 mmol) were sequentially slowly added to a red solution of $RhCl(PPh_3)_3$ (1.04 g, 1.12 mmol) in dichloromethane (35 mL). The reaction mixture was stirred at room temperature for 10 min to yield a red solution. The solvent volume was reduced to approximately 5 mL under vacuum, and the mixture was purified by column chromatography on silica gel (eluent: dichloromethane/acetone = 20:1) to afford complex **2b** as a red solid. Yield: 1.04 g, 81%. ^{1}H NMR (600.1 MHz, CD_2Cl_2): δ 13.08 (s, 1H, C^7H), 11.50 (d, J(HP) = 21.48 Hz, 1H, C^1H), 8.07 (s, 1H, C^3H), 6.88–7.80 (45H, Ph), 2.20 (m, 2H, $C^{10}H$), 1.47 (tt, apparent quint, J(HH) = 7.31 Hz, J(HH) = 7.31 Hz, 2H, C^9H), 1.36 ppm (t, J(HH) = 7.31 Hz, 2H, C^8H). ^{31}P NMR (242.9 MHz, CD_2Cl_2): δ 28.70 (dd, J(PRh) = 108.51 Hz, J(PP) = 5.87 Hz, RhPPh_3), 9.14 ppm (t, J(PP) = 5.87 Hz, CPPh_3). ^{13}C NMR (150.5 MHz, CD_2Cl_2, plus ^{13}C-dept 135 , ^{1}H-^{13}C HSQC and ^{1}H-^{13}C HMBC): δ 261.20 (br, C^7), 239.86 (dt, J(CRh) = 35.44 Hz, J(CP) = 10.37 Hz, C^1), 195.42 (s, C^5), 187.62 (ddt, J(CRh) = 29.21 Hz, J(CP) = 25.20 Hz, J(CP) = 4.83 Hz, C^4), 178.70 (s, C^6), 157.32

(d, J(PC) = 26.58 Hz, C^3), 130.20–134.95 (Ph), 129.41 (ddt, J(CP) = 58.75 Hz, J(CRh) = 8.59 Hz, J(CP) = 4.48 Hz, C^2), 127.80–128.45 (Ph), 119.00 (d, J(CP) = 87.27 Hz, Ph), 32.50 (s, C^8), 31.46 (s, C^9), 28.42 ppm (s, C^{10}). Anal. Calcd (%) for $C_{64}H_{54}BClF_4P_3Rh$: C, 67.36; H, 4.77. Found: C, 67.72; H, 4.89.

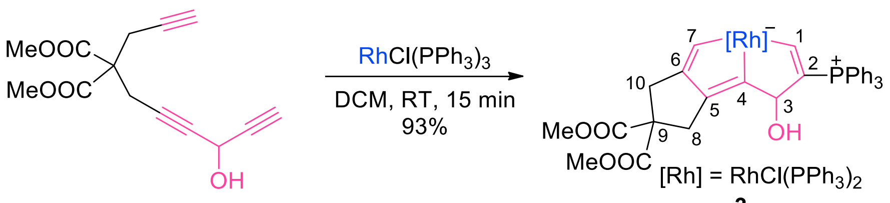

Synthesis and characterization of complex 3: A dichloromethane solution (5 mL) of **1a** (786 mg, 3.00 mmol) was slowly added to a red solution of $RhCl(PPh_3)_3$ (2.73 g, 2.95 mmol) in dichloromethane (90 mL). The reaction mixture was stirred at room temperature for 15 min to yield a green solution. The solvent volume was evaporated under vacuum to approximately 5 mL, and the mixture was washed with hexane (3 × 100 mL) to afford complex **3** as a yellow solid. Yield: 3.26 g, 93%. ^1H NMR (600.1 MHz, CD_2Cl_2): δ 10.27 (d, J(HP) = 29.75 Hz, 1H, C^1H), 6.87–8.20 (45H, Ph), 6.53 (s, 1H, C^7H), 3.57 (s, 3H, $COOCH_3$), 3.54 (s, 3H, $COOCH_3$), 3.45 (d, J(HH) = 8.95 Hz, 1H, C^3H), 2.35 (d, J(HH) = 16.47 Hz, 1H, $C^{10}H$), 2.27 (d, J(HH) = 16.47 Hz, 1H, $C^{10}H$), 2.01 (d, J(HH) = 17.02 Hz, 1H, C^8H), 1.78 (d, J(HH) = 17.02 Hz, 1H, C^8H), 0.17 ppm (d, J(HH) = 8.95 Hz, 1H, OH). ^{31}P NMR (242.9 MHz, CD_2Cl_2): δ 33.33 (ddd, J(PP) = 431.55 Hz, J(PRh) = 123.37 Hz, J(PP) = 5.47 Hz, RhPPh_3), 31.05 (ddd, J(PRh) = 431.55 Hz, J(PP) = 123.37 Hz, J(PP) = 5.47 Hz, RhPPh_3), 8.00 ppm (dt, J(PRh) = 11.00 Hz, J(PP) = 5.47 Hz, CPPh_3). ^{13}C NMR (150.9 MHz, CD_2Cl_2, plus ^{13}C DEPT-135, ^1H-^{13}C HSQC and ^1H-^{13}C HMBC): δ 221.27 (m, C^1), 174.08 (s, $COOCH_3$), 173.27 (s, $COOCH_3$), 167.43 (ddt, J(CRh) = 30.24 Hz, J(CP) = 22.91 Hz, J(CP) = 7.00 Hz, C^4), 154.85 (s, C^6), 153.49 (m, C^7), 145.57 (s, C^5), 127.32–136.42 (Ph), 122.91 (d, J(PC) = 46.59 Hz, C^2), 122.89 (d, J(PC) = 84.89 Hz, Ph), 80.32 (d, J(PC) = 25.69 Hz, C^3), 64.39 (s, C^9), 52.90(s, $COOCH_3$), 52.70 (s, $COOCH_3$), 39.06 (s, C^{10}), 36.01 ppm (s, C^8). Anal. Calcd (%) for $C_{68}H_{59}ClO_5P_3Rh$: C, 68.78; H, 5.01. Found: C, 68.67; H, 5.23.

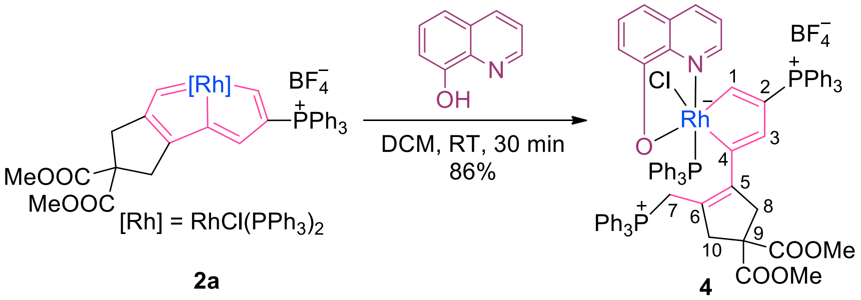

Synthesis and characterization of complex 4: A mixture of **2a** (300 mg, 0.239 mmol) and 8-hydroxyquinoline (104 mg, 0.716 mmol) was stirred at RT in dichloromethane (10 mL) for 30 min to yield a yellow solution. The solvent volume was reduced to approximately 5 mL under vacuum and the mixture was washed with Et_2O (3 × 30 mL) to afford complex **4** as a yellow solid. Yield: 288 mg, 86%. ^{1}H NMR (600.1 MHz, CD_2Cl_2): δ 9.96 (d, J(HP) = 18.51 Hz, 1H, C^1*H*), 6.61–8.07 ppm (51H, Ph and C_9H_6NO), 4.21 (s, 1H, C^3*H*), 3.71 (dd, J (HH) = 15.20 Hz, J (HP) = 15.20 Hz, 1H, C^{10}*H*), 3.67 (s, 3H, COOC*H*$_3$), 3.41(s, 3H, COOC*H*$_3$), 2.09 (dd, J (HH) = 13.79 Hz, J (HP) = 13.79 Hz, 1H, C^7*H*), 1.63 (s, 2H, C^8*H*), 1.26 (dd, J (HH) = 14.32 Hz, J (HP) = 14.32 Hz, 1H, C^7*H*), 0.55 (dd, J(HH) = 17.17 Hz, J(HP) = 9.55 Hz, 1H, C^{10}*H*). ^{31}P NMR (161.9 MHz, CD_2Cl_2): δ 24.65 (d, J(PRh) = 168.18 Hz, Rh*P*Ph$_3$), 14.31 (s, C^7*P*Ph$_3$), 6.75 ppm (d, J(PRh) = 11.24 Hz, C^2*P*Ph$_3$). ^{13}C NMR (150.9 MHz, CD_2Cl_2, plus ^{1}H-^{13}C HSQC and ^{13}C-dept 135): δ 214.66 (m, C^1), 178.74 (m, C^4), 172.62 (s, *C*OOCH$_3$), 170.50 (s, *C*OOCH$_3$), 168.79 (s, C_9H_6NO), 151.97 (d, J(CRh) = 15.41 Hz, C^5), 126.91–143.4 (Ph and C_9H_6NO), 126.53 (d, J(CP) = 10.39 Hz, C^3), 119.89 (s, C_9H_6NO), 119.45 (d, J(CP) = 87.55 Hz, Ph), 119.33 (d, J(CP) = 62.07 Hz, C^2), 116.49 (d, J(CP) = 85.02 Hz, Ph), 115.00 (s, C_9H_6NO), 109.30 (d, J(CP) = 9.01 Hz, C^6), 106.20 (s, C_9H_6NO), 55.21 (s, C^9), 51.81 (s, COO*C*H$_3$), 51.75 (s, COO*C*H$_3$), 44.34 (s, C^{10}), 42.30 (s, C^8), 23.79 ppm (d, J(CP) = 53.03 Hz, C^7). Anal. Calcd (%) for $C_{77}H_{65}BClF_4NO_5P_3Rh$: C, 65.94; H, 4.67; N, 1.00. Found: C, 66.01; H, 4.57; N, 0.65.

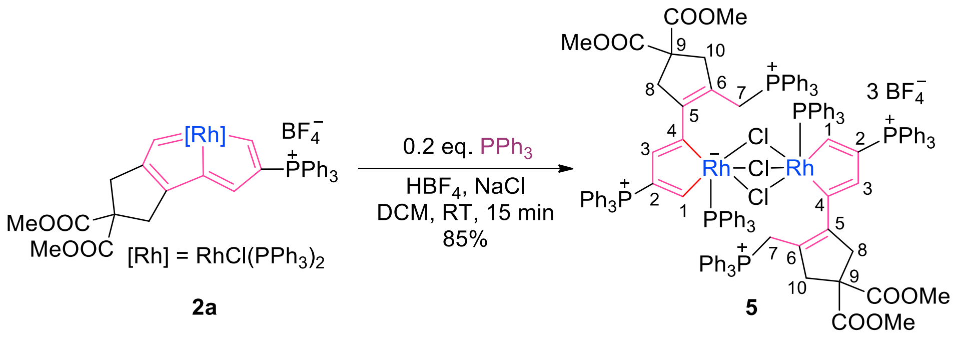

Synthesis and characterization of complex 5: A mixture of **2a** (450 mg, 0.358 mmol) and PPh_3 (18.6 mg, 0.071 mmol) was stirred at RT in dichloromethane (15 mL) for 15 min to yield a yellow

solution. The solvent volume was reduced to approximately 5 mL under vacuum, and the mixture was washed with Et_2O (3 × 30 mL) to afford complex **5** as a yellow solid. Yield: 401 mg, 85%. ^1H NMR (400.1 MHz, CD_2Cl_2): δ 9.18 (d, *J*(HP) = 15.99 Hz, 2H, C^1H), 6.36–7.84 (90H, Ph) , 4.30 (dd, *J* (HH) = 15.20 Hz, *J* (HP) = 15.20 Hz, 2H, C^7H), 4.21 (s, 2H, C^3H), 3.83(s, 6H, $COOCH_3$), 3.53(s, 6H, $COOCH_3$), 3.47 (br, 2H, $C^{10}H$), 2.44 (d, *J* (HH) = 17.14 Hz, 2H, C^8H), 2.07 (d, *J* (HH) = 17.14 Hz, 2H, C^8H) , 1.49 (dd, *J* (HH) = 15.20 Hz, *J* (HP) = 15.20 Hz, 2H, C^7H), 1.01 (dd, *J* (HH) = 16.77 Hz, *J* (HP) = 9.72 Hz, 2H, $C^{10}H$). ^{31}P NMR (161.9 MHz, CD_2Cl_2): δ 25.54 (d, *J*(PRh) = 167.25 Hz, $RhPPh_3$), 15.19 (s, C^7PPh_3), 7.64 ppm (d, *J*(PRh) = 12.0 Hz, C^2PPh_3). ^{13}C NMR (100.6 MHz, CD_2Cl_2, plus ^1H-^{13}C HSQC and ^{13}C-dept 135): δ 198.53 (dd, *J*(CP) = 31.44 Hz, *J*(CP) = 13.77 Hz, C^1), 176.80 (m, C^4), 172.61 (s, $COOCH_3$), 171.28 (s, $COOCH_3$), 151.23 (d, *J*(CRh) = 9.70 Hz, C^5), 130.07–135.91 (Ph), 129.66 (d, *J*(CP) = 21.83 Hz, C^3), 127.83–128.26 (Ph), 122.78 (d, *J*(CP) = 67.52 Hz, C^2), 119.10 (d, *J*(CP) = 88.40 Hz, Ph), 117.46 (d, *J*(CP) = 84.92 Hz, Ph), 113.99 (d, *J*(CP) = 9.61 Hz, C^6), 56.59 (s, C^9), 53.17 (s, $COOCH_3$), 52.95 (s, $COOCH_3$), 44.43 (s, C^{10}), 43.28 ppm (s, C^8), 26.04 ppm (d, *J*(CP) = 51.36 Hz, C^7). Anal. Calcd (%) for $C_{136}H_{118}B_3Cl_3F_{12}O_8P_6Rh_2$: C, 61.90; H, 4.51. Found: C, 62.05; H, 4.48.

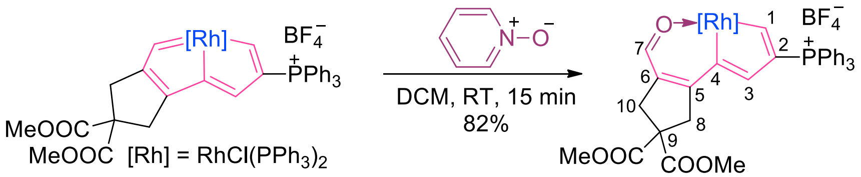

2a **6**

Synthesis and characterization of complex 6: A mixture of **2a** (325 mg, 0.258 mmol) and Pyridine-N-Oxide (73.7 mg, 0.775 mmol) was stirred at RT in dichloromethane (10 mL) for 15 min to yield a blue solution. The solvent volume was reduced to approximately 3 mL under vacuum, and the mixture was purified by column chromatography on silica gel (eluent: dichloromethane/acetone = 20:1) to afford complex **6** as a blue solid. Yield: 269 mg, 82%. ^1H NMR (400.1 MHz, CD_2Cl_2): δ 9. 73 (d, *J*(HP) = 16.11 Hz, 1H, C^1H), 8.28 (s, 1H, C^7H), 6.82–7.84 (45H, Ph), 6.21 (s, 1H, C^3H), 3.74 (s, 6H, $COOCH_3$), 2.86 (s, 2H, C^8H), 2.74 ppm (s, 2H, $C^{10}H$). ^{31}P NMR (161.9 MHz, CD_2Cl_2): δ 26.15 (d, *J*(PRh) = 108.92 Hz, $OsPPh_3$), 8.25 ppm (d, *J*(PRh) = 14.05 Hz, $CPPh_3$). ^{13}C NMR (100.6 MHz, CD_2Cl_2, plus ^{13}C-dept 135, ^1H-^{13}C HSQC and ^1H-^{13}C HMBC): δ 215.90 (m, C^1), 193.06 (s, C^7), 173.99 (m, C^4), 171.16 (s, $COOCH_3$), 157.65 (s, C^5), 144.27 (d, *J*(CP) = 22.03 Hz, C^3), 133.91–135.17 (Ph), 131.53 (s, C^6), 128.30–131.00 (Ph), 123.15 (d, *J*(CP) = 72.70 Hz, C^2), 119.14 (d, *J*(CP) = 88.78 Hz, C^2), 56.20 (s, C^9), 53.63 (s, $COOCH_3$), 41.73 (s, C^8), 39.35 ppm (s, C^{10}). Anal. Calcd (%) for $C_{68}H_{58}BClF_4O_5P_3Rh$: C, 64.14; H, 4.59. Found: C, 63.82; H, 4.40.

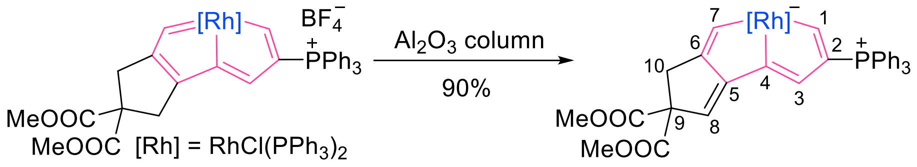

2a **7**

Synthesis and characterization of complex 7: Complex **2a** (300 mg, 0.239 mmol) was dissolved in dichloromethane (5 mL) and put on a neutral alumina column. The red starting material turned a yellow color on the alumina surface. The yellow fraction was eluted with acetone, and the solvent was evaporated in vacuo to afford complex **7** as a yellow solid. Yield: 251 mg, 90%. ^{1}H NMR (500.2 MHz, CD_2Cl_2): δ 10.18 (dd, *J*(HP) = 27.54 Hz, *J*(HP) = 3.30 Hz, 1H, C^1H), 6.88–7.61 (45H, Ph), 6.53 (s, 1H, C^7H), 5.77 (s, 1H, C^3H), 4.68 (s, 1H, C^8H), 3.51 (s, 6H, $COOCH_3$), 2.29 (s, 2H, $C^{10}H$). ^{31}P NMR (202.5 MHz, CD_2Cl_2): δ 32.27 (d, *J*(PRh) = 117.85 Hz, Rh*P*Ph_3), 3.89 ppm (s, C*P*Ph_3). Unfortunately, ^{13}C NMR characterization was failed because of the poor solubility of **7**. Anal. Calcd (%) $C_{68}H_{57}ClO_4P_3Rh$: C, 69.84; H, 4.91. Found: C, 69.88; H, 5.19.

Supplemental References

Becke, A.D. (1993). Density-Functional Thermochemistry. III. The Role of Exact Exchange. J. Chem. Phys. *98*, 5648–5652.

Chen, Z., Wannere, C.S., Corminboeuf, C., Puchta, R., and Schleyer, P.v.R. (2005). Nucleus-Independent Chemical Shifts (NICS) as an Aromaticity Criterion. Chem. Rev. *105*, 3842–3888.

Dolomanov, O.V., Bourhis, L.J., Gildea, R.J., Howard, J.A.K., and Puschmann, H. (2009). OLEX2: A Complete Structure Solution, Refinement and Analysis Program. J. Appl. Cryst. *42*, 339–341.

Fallah-Bagher-Shaidaei, H., Wannere, C.S., Corminboeuf, C., Puchta, R., and Schleyer, P.v.R. (2006). Which NICS Aromaticity Index for Planar π Rings Is Best? Org. Lett. *8*, 863–866.

Frisch, M. J., et al. (2013). Gaussian 09, Revision D.01 (Gaussian: Wallingford CT).

Geuenich, D., Hess, K., Köhler, F., and Herges, R. (2005). Anisotropy of the Induced Current Density (ACID), a General Method to Quantify and Visualize Electronic Delocalization. Chem. Rev. *105*, 3758–3772.

Glendening, E.D., Badenhoop, J.K., Reed, A.E., Carpenter, J.E., Bohmann, J.A., Morales, C.M., Landis, C.R., and Weinhold, F. (2013). NBO 6.0 (Theoretical Chemistry Institute, University of Wisconsin, Madison, WI).

Hay, P.J., and Wadt, W.R. (1985). Ab Initio Effective Core Potentials for Molecular Calculations. Potentials for K to Au Including the Outermost Core Orbitals. J. Chem. Phys. *82*, 299–310.

Huzinaga, S. (1984). Gaussian Basis Sets for Molecular Calculations (Elsevier, Amsterdam).

Lee, C., Yang, W., and Parr, R.G. (1988). Development of the Colle-Salvetti Correlation-Energy Formula into a Functional of the Electron Density. Phys. Rev. B *37*, 785–789.

Miehlich, B., Savin, A., Stoll, H., and Preuss, H. (1989). Results Obtained with the Correlation Energy Density Functionals of Becke and Lee, Yang and Parr. Chem. Phys. Lett. *157*, 200–206.

Schleyer, P.v.R., Maerker, C., Dransfeld, A., Jiao, H., and Hommes, N.J.R.v.E. (1996). Nucleus-Independent Chemical Shifts: A Simple and Efficient Aromaticity Probe. J. Am. Chem. Soc. *118*, 6317–6318.

Sheldrick, G.M. (2015). Crystal Structure Refinement with SHELXL. Acta Cryst. *C71*, 3–8.

Sheldrick, G.M. (2015). SHELXT-Integrated Space-Group and Crystal-Structure Determination. Acta Cryst. *A71*, 3–8.

Wiberg, K.B. (1968). Application of the Pople-Santry-Segal CNDO Method to the Cyclopropylcarbinyl and Cyclobutyl Cation and to Bicyclobutane. Tetrahedron *24*, 1083–1096.
